# Interaction of *Saccharomyces boulardii* with *Salmonella enterica* Serovar Typhimurium Protects Mice and Modifies T84 Cell Response to the Infection

**DOI:** 10.1371/journal.pone.0008925

**Published:** 2010-01-27

**Authors:** Flaviano S. Martins, Guillaume Dalmasso, Rosa M. E. Arantes, Anne Doye, Emmanuel Lemichez, Patricia Lagadec, Veronique Imbert, Jean-François Peyron, Patrick Rampal, Jacques R. Nicoli, Dorota Czerucka

**Affiliations:** 1 Team 4: Inflammation, Cancer, Cancer Stem Cells, Unité INSERM U895, C3M: Centre Méditerranéen de Médecine Moléculaire, Nice, France; 2 Team 6: Toxines microbiennes dans la relation hôte-pathogènes, Unité INSERM U895, C3M: Centre Méditerranéen de Médecine Moléculaire, Nice, France; 3 Université de Nice-Sophia Antipolis, UFR Médecine, IFR50, Faculté de Médecine, Nice, France; 4 Service de Pédiatrie, Centre Hospitalier Universitaire de Nice, Hôpital de l'Archet, Nice, France, Centre Hospitalier Universitaire de Nice, Hôpital de l'Archet, Nice, France; 5 Service d'Hématologie Clinique, Centre Hospitalier Universitaire de Nice, Hôpital de l'Archet, Nice, France; 6 Service de Bactériologie, Centre Hospitalier Universitaire de Nice, Hôpital de l'Archet, Nice, France; 7 Centre Hospitalier Princesse Grace, Service d'Hépato-Gastro-Entérologie, Monaco, Monaco; 8 Departamento de Microbiologia, Instituto de Ciências Biológicas, Universidade Federal de Minas Gerais, Belo Horizonte, Minas Gerais, Brazil; 9 Departamento de Patologia Geral, Instituto de Ciências Biológicas, Universidade Federal de Minas Gerais, Belo Horizonte, Minas Gerais, Brazil; 10 Departamento de Pediatria, Faculdade de Medicina, Universidade Federal de Minas Gerais, Belo Horizonte, Minas Gerais, Brazil; Charité-Universitätsmedizin Berlin, Germany

## Abstract

**Background:**

*Salmonella* pathogenesis engages host cells in two-way biochemical interactions: phagocytosis of bacteria by recruitment of cellular small GTP-binding proteins induced by the bacteria, and by triggering a pro-inflammatory response through activation of MAPKs and nuclear translocation of NF-κB. Worldwide interest in the use of functional foods containing probiotic bacteria for health promotion and disease prevention has increased significantly. *Saccharomyces boulardii* is a non-pathogenic yeast used as a probiotic in infectious diarrhea.

**Methodology/Principal Findings:**

In this study, we reported that *S. boulardii* (Sb) protected mice from *Salmonella enterica* serovar Typhimurium (ST)-induced death and prevented bacterial translocation to the liver. At a molecular level, using T84 human colorectal cancer cells, we demonstrate that incubation with Sb before infection totally abolished *Salmonella* invasion. This correlates with a decrease of activation of Rac1. Sb preserved T84 barrier function and decreased ST-induced IL-8 synthesis. This anti-inflammatory effect was correlated with an inhibitory effect of Sb on ST-induced activation of the MAPKs ERK1/2, p38 and JNK as well as on activation of NF-κB. Electron and confocal microscopy experiments showed an adhesion of bacteria to yeast cells, which could represent one of the mechanisms by which Sb exerts its protective effects.

**Conclusions:**

Sb shows modulating effects on permeability, inflammation, and signal transduction pathway in T84 cells infected by ST and an *in vivo* protective effect against ST infection. The present results also demonstrate that Sb modifies invasive properties of *Salmonella*.

## Introduction

In human, *Salmonella* spp. is responsible for over one billion infections annually, with consequences ranging from self-limiting gastroenteritis to typhoid fever. To initiate disease, *Salmonella* first adheres to and then induces its own uptake into intestinal epithelial cells through a specialized mechanism involving injection of virulence factors into host cells by a type III protein secretion system (TTSS). This activates host signaling pathways involved in actin cytoskeleton rearrangements leading to bacterial uptake [1], [2]. Indeed, *Salmonella*-induced epithelial cell signaling leads to reorganization of the host cytoskeleton, membrane ruffles at the point of bacterium-cell contact leading to internalization of bacteria [3]. Besides, the hijacking of host signaling pathways by *Salmonella* triggers an inflammatory response and the release of inflammatory mediators, such as the interleukin (IL)-8 chemokine [4] that is responsible for recruitment and transepithelial migration of polymorphonuclear leucocytes (PMN) [5]–[7], a specific clinical feature of salmonellosis. Maximal IL-8 amounts are generated by a combination of three different mechanisms: derepression of the gene promoter, transcriptional activation by nuclear factor κB (NF-κB) and c-jun-NH_2_ terminal kinase (JNK) pathways and stabilization of the mRNA by the p38 mitogen-activated protein kinase (MAPK) pathway [8], [9].

In recent years, worldwide interest for the use of functional foods containing probiotic microorganisms for health promotion and disease prevention has increased significantly. According to the Food and Agriculture Organization and the World Health Organization, a probiotic is “a live microorganism which, when administered in adequate amounts, confers a health benefit to the host” [10]. Lyophilized *Saccharomyces boulardii* is a probiotic yeast used worldwide for the prevention and treatment of a variety of diarrheal diseases [11]. In the case of infectious diarrhea, administration of *S. boulardii* to animals provides protection against intestinal lesions caused by several diarrheal pathogens [12]. The mechanisms by which *S. boulardii* exerts its protective effects are diverse, including proteolytic cleavage of *Clostridium difficile* toxins A and B [13], [14], inhibition of cholera toxin stimulated cAMP production [15], binding and elimination of cholera toxin [16], and interference on bacterial-stimulated cellular signaling pathways [17]–[20].

Given the growing interest in the field of probiotic in medical areas, the purpose of this study was to investigate, using an *in vivo* murine model of infection, the protective effect of *S. boulardii* and, to dissect *in vitro* on T84 human colorectal cancer cells the molecular mechanisms mediating *S. boulardii* protection. Our study revealed that *S. boulardii* increased survival of *S.* Typhimurium infected mice and prevented bacterial translocation to the liver. Cellular studies demonstrated that *S. boulardii* decreased the ability of *S.* Typhimurium to invade cells and prevented the secretion of IL-8 through inhibition of the MAPK and NF-κB signaling pathways.

## Results

### Lyophilized *S. boulardii* Decreases Mortality and Prevents Bacterial Translocation to the Liver in Mice Challenged with *S.* Typhimurium

**Figure 1:**
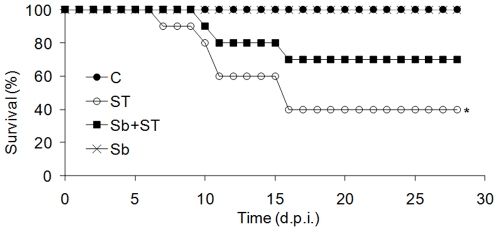
*S. boulardii* decreases *S.* Typhimurium induced death of mice. Survival (%) of mice treated daily (Sb+ST) or not (ST) with *S. boulardii* during 10 days before challenged with 10^4^ *S.* Typhimurium. (C) control (not-treated, not-challenged mice), (Sb) mice treated with *S. boulardii* without bacterial challenge. N = 10. *Indicates statistical difference in relation to control (not treated) group. d.p.i., days post infection.

**Figure 2:**
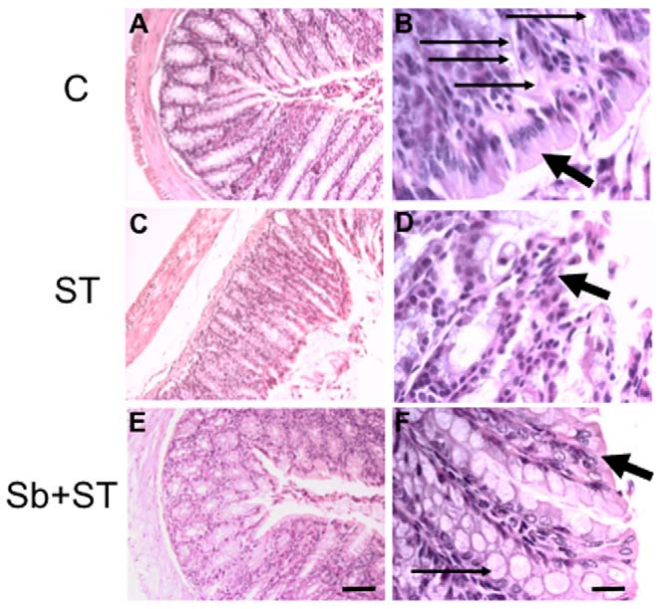
*S. boulardii* protects colon against *S*. Typhimurium lesions. Representative histopathological aspects of large intestine sections in control (A, B); ST (C, D), and Sb + ST (E, F) groups. Notice the integrity of surface colonocytes in B and F (large arrows) contrasting with edema (C), the loss of integrity of epithelial layer and reactive changes of the colonocytes (D, large arrows). Shortening of mucosa length (C) and decrease in goblet cells (D) were observed in the ST group, which were preserved in control (A, B) and in Sb + ST (E, F, thin arrows). H&E, Bar  = 20 µm (A, C, E) and 2 µm (B, D, F). C (control), ST (*S*. Typhimurium), Sb (*S. boulardii*).

Results presented in figure 1 show that an infection of NIH mice with *S*. Typhimurium (ST) killed 60% of mice by 16 days. Oral treatment with *S. boulardii* (Sb) significantly increased (P<0.05) survival from 40% (ST group) to 70% (Sb-treated + ST group). This protective effect was next assessed at the histological level. Mice were sacrificed and tissue samples from intestine were prepared for histological analysis. In control mice (Fig. 2, A and B) and in the Sb + ST group of mice (Fig. 2, E and F) we clearly observed the structural integrity of surface colonocytes contrasting with the loss of integrity of epithelial layer and reactive changes of the colonocytes in ST group (Fig. 2, C and D). Using a germ-free mice model, we have observed that this anti-inflammatory effect is not due to a decrease of ST number by Sb or an ability of the yeast to kill the bacteria (Supporting Figure S1).

**Figure 3:**
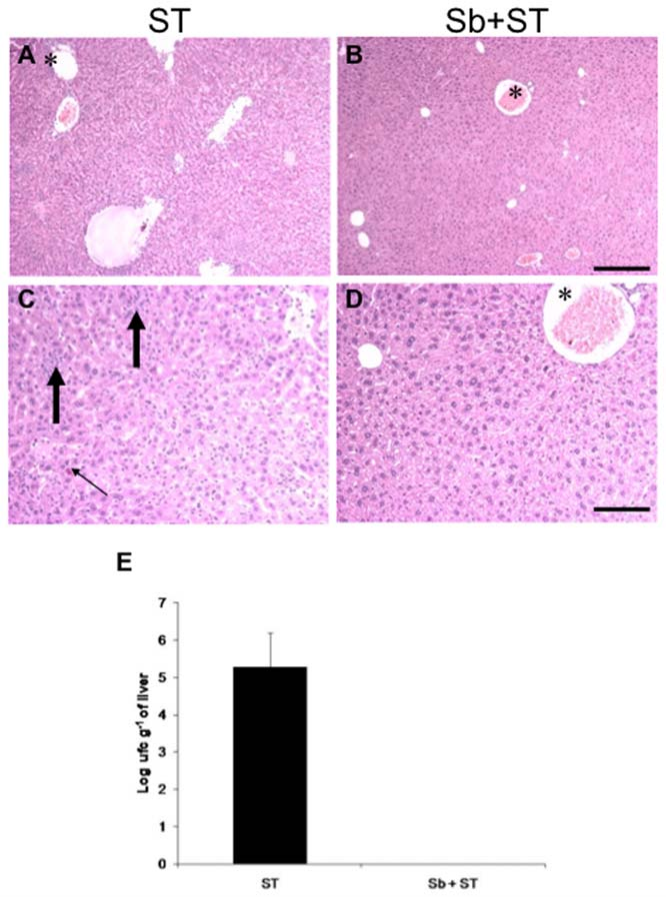
*S. boulardii* protects liver against *S.* Typhimurium lesions. Representative aspects of liver in ST (A, C) and Sb + ST (B, D) groups showing the portal spaces (*) and its surrounding parenchyma. In A and C there are diffuse small foci of inflammatory cells (large arrows) and degenerative changes (thin arrow) contrasting with architectural preservation and absence of significant changes in liver parenchyma of treated group (B, D). H&E, Bar  = 20 µm (A, B) and 10 µm (C, D). (E) *S. boulardii* prevents *S.* Typhimurium translocation to the liver. Translocation of *S.* Typhimurium to liver in experimental (Sb + ST) or control (ST) conventional mice challenged intragastrically with 4.0 log CFU of the bacteria. Determination was performed ∼10 days after challenged (beginning of mortality). Vertical bars represent standard deviations of the means. N = 10. ST (*S*. Typhimurium), Sb (*S. boulardii*).

Data presented in Figure 3 showed that ST group animals presented evidences of bacterial translocation to liver. This is evidenced by the higher cellularity, presence of inflammatory cells and, degenerative changes of hepatocytes. In the Sb-treated group, the pathogenic bacterial infection did not induce significant inflammatory changes in the liver parenchyma, which preserves its normal lobular architecture. This observation was confirmed by measure of the bacterial load in the liver: 5.3±0.9 log colony forming units (CFU) g^−1^ of organ in the ST-group, while no bacteria could be detected in the group previously treated with Sb (Fig. 3E).

### Lyophilized *S. boulardii* Preserves Barrier Function of *S.* Typhimurium-Infected T84 Cells

**Figure 4:**
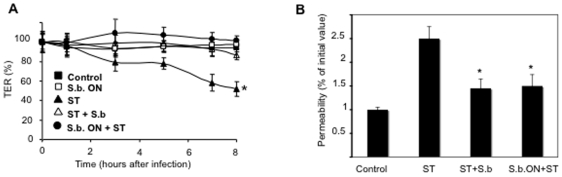
*S. boulardii* preserves barrier function of *S.* Typhimurium-infected T84 cells. (A) Transepithelial electrical resistance (TER) were measured at different time in: (Control) control T84 monolayers, (S.b. ON) monolayers incubated with *S. boulardii* alone (overnight), (ST) monolayers infected with ST alone, (ST + S.b.) monolayers infected with ST in the presence of *S. boulardii* added at the same time, or monolayers incubated overnight with *S. boulardii* prior the infection (S.b. ON + ST). The TER values are displayed as percentages of initial values. (n = 5). An asterisk denotes significantly different versus control cells (P<0.05). (B) Permeability of T84 monolayers to FITC-dextran was determined in monolayers exposed to ST alone or to ST and *S. boulardii*. Graph represents percentages of variation of permeability as compared to initial value (t = 30 min) of each monolayers. (n = 2). An asterisk denotes significantly different versus ST infected cells (P<0.05). The data are expressed as means ± SEMs.

To evaluate the cellular effects of Sb on intestinal epithelium resistance, we infected human T84 cell monolayer by ST. Results in figure 4A show that ST infection diminished gradually the electrical resistance (TER) of monolayers. A significant decrease in TER (25%, P<0.05) was already recorded at 3 h after infection when compared to control monolayers. At 8 h post-infection, the resistance dropped almost to 50% (Fig. 4A). When Sb was added either before or at the moment of ST infection, the TER was maintained at the level of uninfected monolayers. Overnight challenge with yeast alone had no effect on TER. The permeability activity of T84 monolayers was also evaluated by measuring the paracellular diffusion of FITC-dextran across T84 monolayers. Data presented in Figure 4B show that at 7 hours after infection, the permeability of T84 monolayers to FITC-dextran increased in the condition of monolayers infected by ST alone. This phenomenon was significantly reduced (P<0.05) in the presence of Sb added either before or at the moment of infection by ST. Altogether these data show that Sb actively protects monolayer barrier function during infection by ST.

### Effect of Lyophilized *S. boulardii* on *S.* Typhimurium Growth, Adhesion and Invasion of T84 Cells

In order to decipher the protective effect of Sb, we first analyzed whether the yeast could inhibit the growth of bacteria. As presented in Table 1 the growth of ST was not affected by Sb (1.62±0.79×108 CFU well^−1^ in ST group, 1.81±0.18×10^8^ CFU well^−1^ in Sb + ST group, and 1.85±0.42×10^8^ CFU well^−1^ in Sb overnight + ST group). Moreover, measurement of *Salmonella* fecal content of gnotobiotic mice either untreated or treated with Sb show that ST is not affected by Sb (Supporting Figure S1). As *Salmonella* adhesion to host cells is the first necessary step for invasion, this parameter was investigated on T84 cells. Data presented in Table 1 show that Sb did not significantly change the number of adherent bacteria to T84 cells. Then, using a classical gentamicin protective assay, we evaluated T84 cell invasion. The number of intracellular bacteria recovered in T84 cells infected by ST alone was 0.3±0.03×10^6^ CFU well^−1^ corresponding to 11.7% of total bacteria. When Sb was applied together with ST the number of intracellular bacteria significantly decreased to a value of 0.2±0.013×10^6^ CFU well^−1^ representing 5.12% of total bacteria. Overnight pre-incubation with Sb further increased the effect as the number of bacteria dropped to 0.016±0.007×10^6^ CFU well^−1^, corresponding to 0.37% of total bacteria. These data clearly demonstrate that Sb can interfere with host cell invasion by ST.

**Table 1:** Effect of *S. boulardii* (Sb) on adhesion and invasion of *S*. Typhimurium in T84 cells.

Cell treatment	Mean no. ± SEM of			% Invasion
	Total bacteria (x 10^8^ CFU/well)	Cell-associated bacteria (x 10^6^ CFU/well)	Intracellular bacteria (x 10^6^ CFU/well)	
*S*. Typhimurium	1.62±0.79	2.25±0.21	0.30±0.030	11.78±0.90
Sb + *S*. Typhimurium	1.81±0.18	3.80±0.52	0.20±0.013	5.12±0.64*
Sb (ON) + *S*. Typhimurium	1.85±0.42	4.56±0.10	0.016±0.007	0.37±0.20*

T84 cells were infected for three hours with ST ATCC 14028 (10^8^ CFU/well) in the presence (or not) of the yeast (10^7^ CFU/well). Invasion was assessed by the gentamicin protection method.

*Indicates statistical difference in relation to *Salmonella* alone infected cells (P<0.05).

(n = 3).

### Analysis of Interactions between *S. boulardii* and *Salmonella*

**Figure 5:**
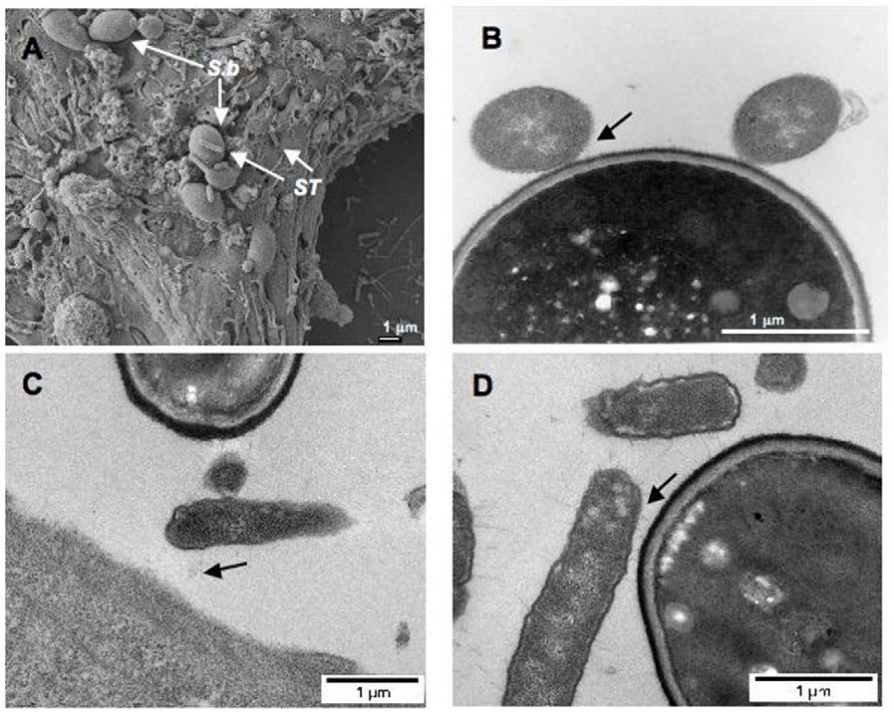
Binding of *S.* Typhimurium (ST) to *S. boulardii* (Sb) cell wall. (A) Scanning electron micrograph of T84 cells infected by ST in the presence of Sb. (B) Transmission electron microscopy. (C, D) Transmission electron micrograph after red ruthenium staining. N = 3. Black arrows show the binding of bacteria to the yeast.

**Figure 6:**
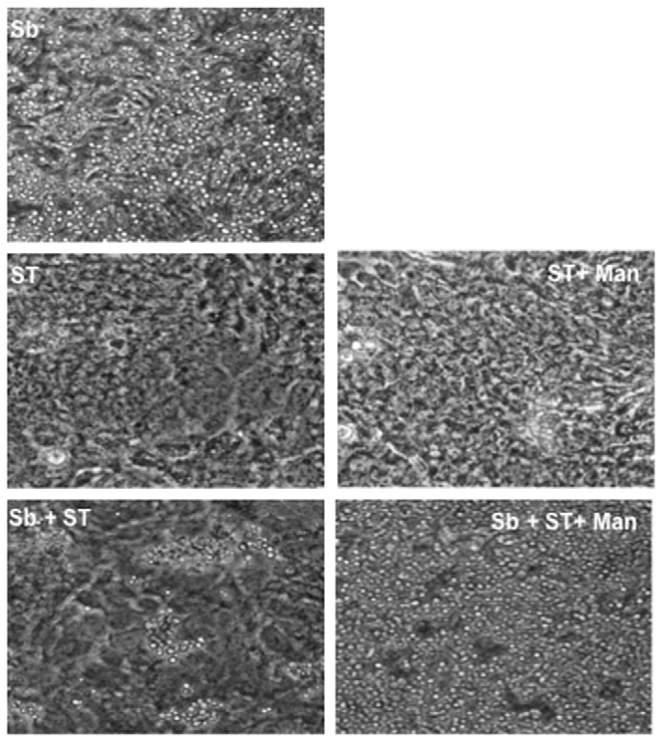
Phase-contrast microscopy showing agglutination between *S. boulardii* (Sb) and *S*. Typhimurium (ST) in the presence and absence of α-D-mannose. ST (*S*. Typhimurium), Sb (*S. boulardii*), Man (α-D-mannose).

To investigate whether ST might bind to yeast cells, electron and confocal microscopy techniques were used. Scanning electron micrograph of T84 cells infected by ST in the presence of Sb shows yeasts on the surface of cell monolayer and ST adhering specifically to yeast cells (Fig. 5A). Sb did not totally coat the monolayer, and thus may explain the fact that the yeast did not totally prevent ST penetrating the T84 cells. Transmission electronic micrograph confirmed that the bacteria can bind intimately to the yeast (Fig. 5B). To investigate more precisely this interaction we used ruthenium red coloration. Ruthenium red is a polyvalent cation that binds a range of highly charged polyanions such as glycoproteins, and thus we could underline adhesion between bacterial pili and the cell wall of Sb (Fig. 5, C and D). Confocal micrographs also confirm binding of ST to Sb cell wall (data not shown). Since *Salmonella* piliated strain adhesion to *Saccharomyces cerevisiae* has been shown to be mannose sensitive [21], we analyzed the effects of mannose in Sb-ST interaction. As shown in Figure 6, agglutination between Sb and ST was inhibited by α-D-mannose suggesting that adhesion is mediated by type I fimbriae.

### Effects of Lyophilized *S. boulardii* on Signaling Pathways Induced upon *S.* Typhimurium Infection

**Figure 7:**
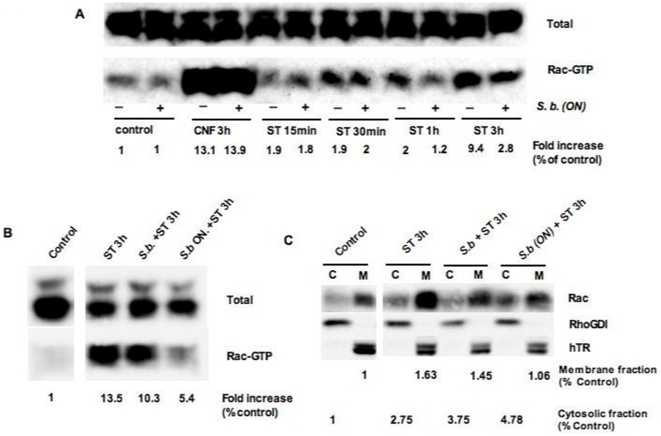
*S. boulardii* prevents Rho-GTPase Rac1 signaling pathway in HeLa cells. (A) Activation of Rac1 in cells infected for various time with *S.* Typhimurium (ST) strain SL1344 in the presence or absence of *S. boulardii* (Sb) (representative of five independent experiments). (B) Activation of Rac1 in cells infected for 3 h by ST alone or ST and Sb added at the same time and in cells exposed overnight to Sb prior infection (representative of two independent experiments). At indicated time, cells were lysed and GTP-bound Rac1 were precipitated from lysates by incubation with GST-PAK, resolved by SDS-PAGE and analyzed by immunoblotting using anti-Rac1 antibody. The control lane contained uninfected cells. CNF1 is a toxin produced by uropathogenic *E. coli* that activate Rac1 [34] and it is used in this study as a positive control. (C) Immunoblots showing the distribution of Rac proteins between membrane (M) and cytosolic (C) fractions in HeLa cells infected with ST for 3 h alone or in the presence of Sb (representative of two independent experiments). Immunoblots were performed with RhaGDI as loading control for cytosolic fraction and human Transferrin Receptor (hTR) was used as loading control for membrane fraction. Densitometry was performed using Multi Gauge software.

The Rac1 GTPase is important for cytoskeleton remodeling during bacteria infections. Kinetics studies performed with ST strain ATTC 14028 did not show significant activation of Rac1 in T84 or Hela cells (data not shown). For this reason the more invasive SL1344 strain was used in these series of experiment. SL1344 strain induced a rapid activation of Rac1 in HeLa cells measured as the amount of its GTP-bound form. Activation was detectable 15 min after the beginning of infection and further increased after 3 h of infection (9 fold, Fig. 7A) The *Escherichia coli* CNF1 toxin, used as a positive control [22], strongly activated Rac1 (13 fold). We next compared two modes of Sb treatment, either by simultaneous addition (curative protocol) or by overnight pre-incubation (preventive protocol). While in the preventive protocol, Sb could interfere with ST-induced Rac1 activation (5.4 vs 13.5 fold, Fig. 7B), only a slight inhibitory effect was detected using the curative protocol (10.3 vs 13.5 fold).

Cell fractionation experiments were performed to visualize activated Rac1 that locates to membranes. ST infection induced a strong membrane localization of Rac1 (1.6 fold) that was significantly decreased in cells pre-incubated with yeast (1.06 fold). Of note, this decrease in membrane associated Rac1 was mirrored by an increase of Rac1 in cytosolic fractions. These two approaches demonstrated that pre-incubation of cells with Sb strongly interfered with Rac1 activation by ST.

### Effect of Lyophilized *S. boulardii* on IL-8 Secretion and Synthesis in *S.* Typhimurium Infected Cells

**Figure 8:**
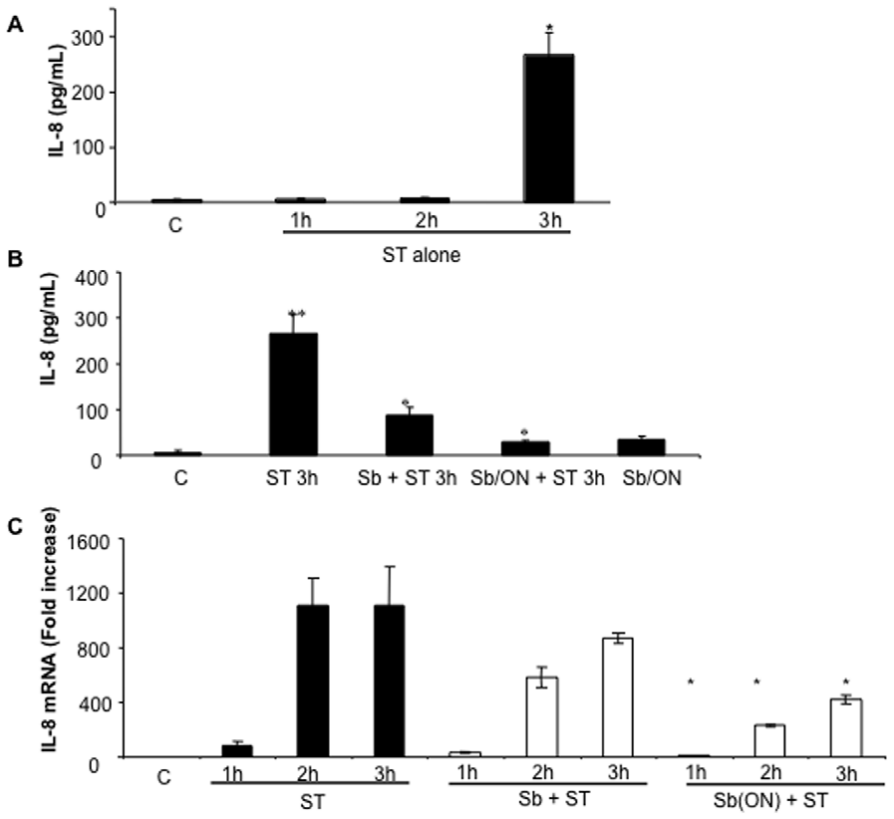
*S. boulardii* prevents IL-8 secretion and synthesis in *S.* Typhimurium infected cells. Pro-inflammatory response of T84 monolayers in response to *S.* Typhimurium infection in the absence (A) or presence (B) of *S. boulardii*. (A) At indicated time of infection, medium was collected and IL-8 content was estimated by ELISA. The asterisk indicates that the value is significantly different from the value for uninfected control cells (P<0.01), as determined by the Bonferroni-Dunn tests. (B) IL-8 content was estimated in the supernatant of T84 cells after 3 h of infection with ST in the presence (curative and preventive protocol) or absence of *S. boulardii*. Errors bars indicate standard deviations. An asterisk denotes significantly different versus ST-infected cells and two asterisks denotes significantly different versus control cells (P<0.05) when compared by the Student's *t*-test. ON, overnight. The data in A and B are expressed as means ± SEMs (n = 8). (C) IL-8 mRNA content was estimated in T84 cells by real time PCR after 1, 2 and 3 h of infection with ST in the presence (curative and preventive protocol) or absence of *S. boulardii*. Errors bars show the SEMs. An asterisk denotes significantly different versus ST-infected cells (P<0.05, n = 6) when compared by the Student's *t*-test. ON, overnight.

We next examined the ability of Sb to reduce or prevent key inflammatory responses following ST infection. Kinetic studies showed that a significant increase in IL-8 production was observed 3 h after ST infection (266.14±41.3 pg/ml versus 4.06±1.56 pg/ml for uninfected cells) (Fig. 8A). Addition of Sb together with ST significantly lowered IL-8 production (86.7±17.6 pg/ml). Moreover, overnight pre-incubation almost abolished IL8 production (29.3±2.7 pg/ml) (Fig. 8B). Sb alone did not induce significant IL-8 release (31.5±5.7 pg/ml). Expression of IL-8 mRNA started one hour after ST infection (78.5 fold) and reached a plateau after 2 and 3 hours (respectively 1110.5 and 1106.4 folds) (Fig. 8C). Simultaneous addition of Sb and ST for 1, 2 or 3 h did not modify IL-8 mRNA content when compared to cells infected by ST alone. However, when cells were pre-incubated with yeast before ST infection, a significant decrease in ST-stimulated IL-8 synthesis was observed (9.5, 4.8 and 2.7 fold decrease, after 1, 2 and 3 h of ST infection, respectively) (Fig. 8C).

### Effect of Lyophilized *S. boulardii* on *S.* Typhimurium-Induced MAPK Activation and NF-κB Signaling Pathway

**Figure 9:**
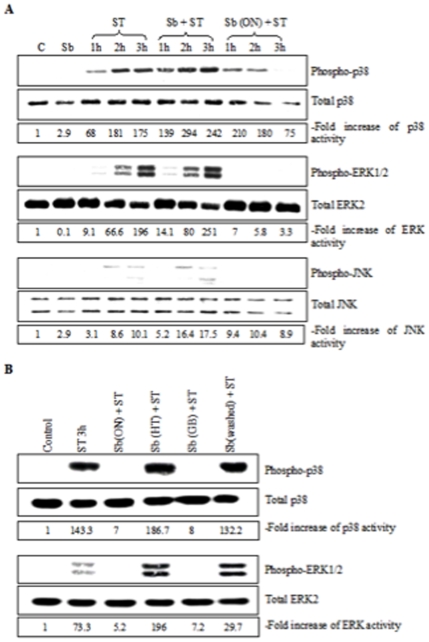
*S. boulardii* influences *S.* Typhimurium-induced MAPK activation (A). (B) Effect of *S. boulardii* (Sb ON), Sb heat treated (Sb HT), Sb glass beads treated (Sb GB), and Sb PBS washed (Sb washed) on MAPK (p-38 and ERK1/2) activation induced by ST in infected T84 cells. Cells were lysed at indicated times after infection. Samples were resolved by SDS-PAGE and analyzed by immunoblotting with anti-phospho-ERK1/2, anti-phospho-p38, and anti-phospho-JNK antibodies. Total ERK, p38 and JNK are shown as loading controls. Control lanes correspond to uninfected T84 cells and T84 cells with Sb. ON, overnight. (C) Samples were resolved by SDS-PAGE and analyzed by immunoblotting with anti-phospho-IκB or anti-IκB antibodies. The control lane corresponds to uninfected T84 cells. Densitometry was performed using Image J software [33]. Western blots shown here are representative of 5 independent experiments.

We next investigated the effect of Sb on different signaling pathways that are known to be involved in IL-8 production in kinetics experiments using phospho-specific antibodies. Blots were probed with antibodies recognizing the non-phosphorylated form of MAPKs to rule out any variation of the total protein amount during the infection procedure. The phosphorylated active-forms of ERK1/2 (p42 and p44), p38 and JNK (p46 and p54) were nearly undetectable in control cells and in cells treated with Sb alone (Fig. 9A). Activation of p38 and ERK1/2 were detectable after 1 h of ST infection and increased after 2 and 3 h. Activation of JNK was not detectable until 2 h of infection. When Sb was simultaneously added with bacteria the level of phosphorylation of the three MAPKs was similar to the levels measured in cells infected by ST-alone. In contrast, pre-incubation of cells with Sb prior to ST infection decreased activation of ERK1/2 and JNK kinases (Fig. 9A). The effect of Sb on p38 activation appeared less pronounced although no more activation could be detected at 3 hs.

We next tried to characterize the mode of Sb's action on MAPK. Before incubation on cells, Sb received different treatments: heat inactivation (HT), glass beads (GB) treatment or PBS washed (Sb were pre-incubated with T84 cells and before *Salmonella*-infection, yeast was removed by several PBS washings). Figure 9B shows that, HT and PBS washing totally abolished the inhibitory effect of Sb on MAPK activation. Thus Sb must be alive and present during infection. By contrast, the inhibitory effect of Sb was maintained when an extract obtained after disruption of the yeast with GB was used. This result suggests that components of yeast cell structure were implicated in the inhibitory effect observed.

**Figure 10:**
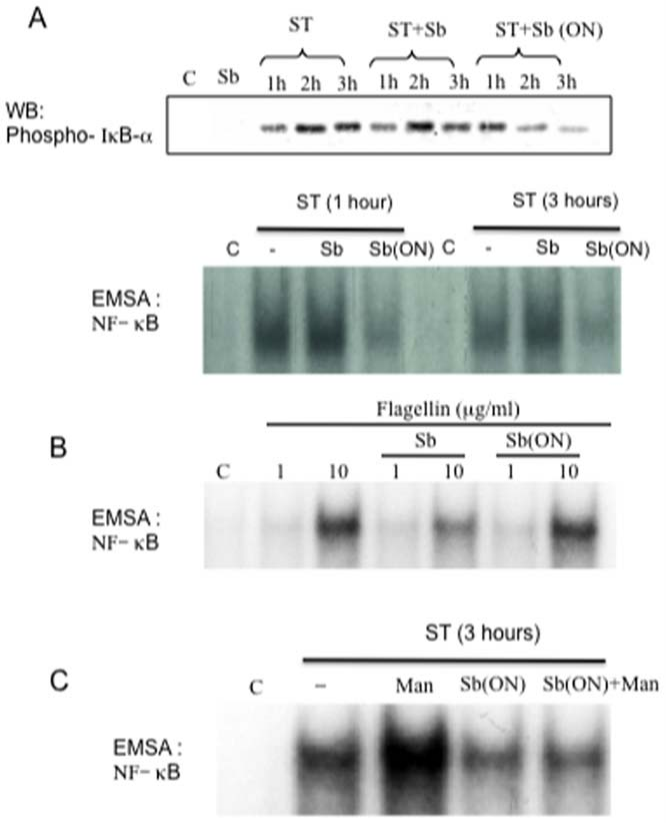
*S. boulardii* influences on *S.* Typhimurium-induced IκB-α phosphorylation and NF-κB signaling pathway. (A) Samples were resolved by SDS-PAGE and analyzed by immunoblotting with anti-phospho-IκB antibodies. Determination of NF-κB binding activity was analyzed by EMSA. (B) *S. boulardii* did not decrease NF-κB binding activity induced by 1 or 10 µg/ml of flagellin. (C) *S. boulardii* induced decrease of NF-κB binding activity is not modified in the presence of α-D-mannose. Western blots and EMSA shown are representative of 5 independent experiments.

We next examined the action of Sb on NF-κB activation. For this, we first evaluated phosphorylation of IκB-α inhibitory subunit, which results in IκB-α proteasomal degradation and translocation of active NF-κB into the nucleus. In T84 cells, ST induced phosphorylation of IκB-α that occurred 1 h after the beginning of infection, increased at 2 h and remained elevated over the course of 3 h (Fig. 10A). ST-induced phosphorylation of IκB-α decreased when cells were pre-incubated overnight with Sb but not when Sb and bacteria were simultaneously applied An EMSA experiment showed a strong NF-κB binding activity 1 h after the beginning of ST infection that remained elevated over 3 h (Fig. 10A). Again, overnight pre-incubation with Sb, but not simultaneous addition completely prevented NF-κB activation. Flagellin represents a major pro-inflammatory determinant of ST that is able to activate NF-κB. As shown on Fig 10B, Sb did not blocks flagellin-induced NF-κB activation. The presence of mannose during infection did not interfere with the inhibitory effect of Sb on ST-induced NF-κB activation (Fig. 10C). This result suggests that inhibition of NF-κB binding activity is independent of ST adhesion on Sb.

## Discussion

A wide range of antibiotics are used to treat human salmonellosis. However, genetic mutations and selective pressure have pushed *Salmonella* spp., as well as other bacteria, to become resistant or multi-resistant to antibiotics [23], [24]. Development of alternative natural processes for the treatment and prevention of gastrointestinal disorders, such as probiotics, has become attractive therapeutic options. In the present work we investigate the effects of the non pathogenic yeast *S. boulardii* on *Salmonella* infection. A protective effect of lyophilized Sb against ST infection has been already described in conventional and gnotobiotic mice [25]. In the present study we have investigated the protective effect of lyophilized Sb against *Salmonella* infection at cellular and molecular levels. Two primordial aspects were identified: Sb affects the invasion property of ST, and it exerts an anti-inflammatory effect.

Among the five cell lineages present in the intestinal epithelium (i.e. enterocytes, M cells, goblet cells, enterochromaffin, Paneth cells), M cells and enterocytes are generally thought to be the most important for bacterial invasion and transcytosis [26]. The relative importance of these two lineages as an entry portal for ST is a matter of debate. After experimental infection with high doses of ST, most bacteria are observed within the M cells in the small intestine, indicating that these cells have a higher capacity to internalize ST as compared to surrounding enterocytes. On the other hand, a lower uptake efficiency of enterocytes for ST may be compensated by its greater numbers, as they greatly outnumber M cells in the small intestine (by >100∶1). Furthermore, the colon is markedly devoid of M cells, yet the colonic mucosa can be efficiently colonized by ST. Cell culture studies on the interaction between ST and intestinal epithelial cells have generally been performed with model that mimic enterocytes, i.e. the human colonic epithelial T84 cells.

Histological data presented in this study show that enterocytes architecture in the colon of infected mice was preserved in the presence of Sb during ST infection. Study performed on filter grown T84 cells confirmed this observation. Translocation of ST across the intestinal epithelium to the liver is greatly attenuated in mice receiving Sb. Incubation of T84 cells with Sb maintained the TER of ST-infected monolayer, suggesting that the presence of the yeast decreased the paracellular permeability of cells exposed to ST.

As demonstrated using the T84 cells, Sb did not affect the number of cell-attached *Salmonella* but significantly decreased the number of intracellular bacteria. The main finding of our study is the demonstration of a physical interaction of Sb with ST that could account for the observed interference with bacterial invasion. In case of cells incubated with yeast and bacteria, the number of “cell associated” bacteria is slightly more elevated that in cells incubated with ST alone. This increase can be explained by observations made using scanning electronic microscopy which revealed few yeasts attached to the monolayer and many bacteria attached to the yeast cell wall. Yeast and adherent bacteria formed aggregates attached to cell monolayer and this probably contributed to the increased number of bacteria depicted in our assay as adherent bacteria. Previous reports have shown that *Salmonella* strains that express type I fimbriae have some affinity to Sb. This affinity is mediated by mannan oligosaccharides of yeast cell wall, and can be inhibited by mannose complexes [27]. The aggregation between yeast and bacteria described in our study was also inhibited by mannose. Transmission electronic micrograph showed that the bacterial pili are in contact with yeast. Microscopic study revealed that the yeast attracts and binds many of the bacteria explaining why a low number of ST alone was observed adhered to the monolayer of T84 cells. Once the yeast attracted the bacteria on its surface, a diminished or inhibited signaling cascade was generated by a lower number of bacteria in direct contact with the host cell. Another consequence of Sb addition during infection was the decrease of *Salmonella*'s cell invasion by more than 50% (5.1% versus 11.8% in *Salmonella* alone infected cells). The invasive properties of *Salmonella* were drastically decreased (and reach a level of 0.3% of the control) in cells that were incubated with yeast prior to infection. However, as depicted on the scanning electron micrograph, *Salmonella* that are in contact with T84 cells can also be visualized in that monolayers exposed to yeast before infection. This indicates that besides adhesion of bacteria to yeast, Sb decreased the invasion property of ST by an additional mechanism.

*Salmonella* internalization is closely associated to injection through the TTSS of effectors proteins that activate the Rho family of GTPases. Recent evidence suggests that only Rac1 and RhoG are indispensable for actin remodeling events that are generated by *Salmonella* spp. during host cell entry [28]. We thus investigated the effect of Sb on Rac1 activation in ST infected cells. In *Salmonella*-infected HeLa cells, activation of Rac1 started within 30 min after infection and increased over 3 h of infection. The active form of Rac1 was significantly decreased when cells were treated overnight with Sb before ST infection. This observation was supported by another set of experiments on Rac-GTP recruitment to the membrane. As demonstrated in this study, the recruitment of the active Rac1 to the membrane is decreased in cells infected in the presence of yeast. This decrease is correlated with an increase of the inactive Rac1 found in the cytosolic fraction. Altogether, these results indicate that the yeast interferes with *Salmonella* induced signaling pathways that are implicated in bacteria internalization. A recent study reported that Sb did not reduce host cell invasion by *Shigella* [20]. This difference may be due in part to the different relationships between these bacteria and the host cell. *Salmonella* spp. utilizes TTSS to directly activate Cdc42 and Rac1, whereas for *Shigella* spp., the TTSS effector proteins do not engage the GTPase directly, but rather they generate signals through activation of src family tyrosine kinase [29].

The fact that Sb reduces *Salmonella* invasion, and induced activation of host Rho GTPase, is of primordial importance in the prevention of pathogenesis, since activation of Rho family is not only implicated in actin cytoskeleton rearrangement but also in modulation of tight junction and induction of inflammatory responses.

The stimulation of Cdc42 triggers several MAPKs pathways including ERK1/2, JNK and p38, which results in the activation of the transcription factors AP-1 and NF-κB. These transcription factors then direct the production of pro-inflammatory cytokines such as IL-8, which stimulate PMN transmigration and the inflammatory response leading to diarrhea. Kinetic studies presented in this report show that after 3 h of infection, *Salmonella* stimulated IL-8 secretion. Secretion of IL-8 was significantly decreased in cells exposed to Sb during infection, but the IL-8 mRNA level was not affected by the presence of the yeast. However, when cells were pre-incubated with yeast before infection, both IL-8 secretion and IL-8 mRNA level were decreased to basal levels. These results suggest that Sb acts at two levels. When added simultaneously with infection, Sb directly acts on the cytokines or the secretory process without affecting the transcription machinery. When added before infection, Sb modifies IL-8 gene transcription. These results suggest an interference of Sb with MAPKs pathways and NF-κB activation. However, data presented in this study reveal that addition of yeast during infection did not modify activation of any MAPKs or NF-κB activation. Conversely, incubation of cells with yeast before infection inhibited activation of ERK1/2 and JNK pathways and, induced a decrease in p38 phosphorylation and IκB. The anti-inflammatory properties of Sb have been reported in the case of infection caused by EHEC [19], *C. difficile* [17] and, recently in the case of *Shigella* [20]. Reduction of IL-8 levels was in each case correlated with inhibition of signaling pathways that play a role in IL-8 production. In the case of *C. difficile* and *Shigella* infection, Sb supernatant also exhibits an anti-inflammatory effect suggesting that soluble factor(s) produced by the yeast is (are) implicated. A fraction containing a small (<1 kDa) heat-stable and water soluble anti-inflammatory molecule, termed *Saccharomyces* anti-inflammatory factor (SAIF), has been identified in the yeast supernatant [30]. This fraction inhibited NF-κB activation by LPS, IL-1β and TNF-α. As reported in our study, the inhibitory effect disappeared after heat treatment of yeast but was present in the supernatant containing yeast cell wall. These results suggest that a heat labile molecule present in the cell wall of the yeast mediates the inhibition of MAPKs.

Concluding, the results presented here clearly demonstrate that *S. boulardii* modifies the invasive properties of *Salmonella*. Addition of yeast during infection exerts a beneficial effect both by reducing by 50% invasion by *Salmonella* and maintaining the barrier function of epithelial monolayer. However, exposure to yeast before infection, significantly amplifies the beneficial effect. The invasion of *Salmonella* is completely abolished and the inflammatory process is stopped. Additionally, exposure to Sb before the infection modulates activation of Rac1, MAPK and NF-κB during infection.

## Materials and Methods

### Microorganisms

Two strains of *Salmonella enterica* serovar Typhimurium (ST) were used in this study. The strain ATCC 14028 was kindly provided by Oswaldo Cruz Foundation (FIOCRUZ), Rio de Janeiro, Brazil. The strain SL1344 was kindly provided by Stéphane Meresse, Centre d'Immunologie de Marseille-Luminy, CNRS-INSERM Université de la Méditerranée, Marseille, France. Bacteria were stored in Luria-Bertani (LB) medium plus 15% glycerol at –80°C and grown in LB broth overnight at 37°C without shaking. Cultures of *S. boulardii* (Sb) were obtained by inoculating a commercial lyophilized preparation of the yeast (Ultra-Levure®, BIOCODEX, France) and growing overnight at 37°C, with shaking, in Halvorston minimal medium with 2% glucose.

### Mice

Conventional 21-day-old NIH mice (Taconic, USA) were used in this work. Water and commercial autoclavable diet (Nuvital, Brazil) were sterilized by steam and administered *ad libitum,* and animals were maintained in an open animal house with controlled lighting. All experimental procedures were carried out according to the standard procedures [31]. The study was approved by the Ethics Committee in Animal Experimentation of the Federal University of Minas Gerais (protocol N° 11/2007).

### Determination of *S. boulardii* Protective Effect *In Vivo*

To investigate the effect of Sb on the mortality induced by ST infection, mice were orally treated, during 10 days, with 0.1 ml containing 10^8^ colony forming units (CFU) of viable Sb or 0.1 ml vehicle (control) and then challenged with 10^4^ CFU ST. Cumulative mortality was accompanied during 28 days post infection (d.p.i).

### Determination of S. Typhimurium Translocation *In Vivo*

To investigate the effect of Sb on ST translocation to liver, mice were treated, during 10 days, with 0.1 ml containing 10^8^ CFU of viable Sb or 0.1 ml vehicle (control) and then challenged with 10^4^ CFU ST. Bacteria translocation was determined after 10 days of challenge, as previously determined [32].

### Histological Analysis

Tissue samples from intestines of sacrificed mice were fixed in buffered 4% formaldehyde and processed for paraffin embedding. The histopathological sections were stained with hematoxylin-eosin (H&E) and examined by a single pathologist, who was unaware of the experimental conditions.

### Cell Lines and Growth Conditions

The human T84 colonic cell line and HeLa cells were obtained from the European Collection of Animal Cell Cultures (Salisbury, England). The T84 culture medium contained a 1∶1 mixture of Dulbecco-Vogt modified Eagle medium and Ham's-F12 medium (DMEM/F12) supplemented with 50 µg ml^−1^ penicillin, 50 µg ml^−1^ streptomycin (Sigma, France), and 4% fetal bovine serum (Hyclone, France). HeLa cells were maintained in DMEM medium with antibiotics and 10% fetal bovine serum (Hyclone, France).

### Infection Procedure *In Vitro*

For infection, T84 cells were seeded into six-well tissue culture plates at 10^6^ cells per well and grown to confluence. Prior to infection, culture medium was changed to medium without serum and antibiotics. Bacteria, grown overnight into LB broth medium, were pelleted by centrifugation, re-suspended in DMEM/F12 medium, and added to cells (10^8^ bacteria well^−1^). After determined times of infection, bacteria were eliminated by several washes with cold sterile phosphate-buffered saline (PBS) and cell monolayers were frozen in liquid nitrogen and conserved at −80°C for protein determination. When infection was performed in the presence of yeast (10^7^ yeasts well^−1^), cells were pre-incubated overnight with Sb and then infected by ST (preventive protocol) or received the yeast simultaneously (co-infection) with the infection (curative protocol). The yeast-to-bacterium ratio did not modify intestinal cell viability. For infection of filter-grown cells, bacteria (10^7^ bacteria well^−1^) and yeast (10^6^ bacteria well^−1^) were added to the apical compartment.

### Determination of Epithelial Monolayer Resistance and Permeability

T84 cells were grown on collagen-coated, 0.33-cm^2^ porous filter membranes (3-mm-diameter pores; Costar; Poly Lab. Paul Block, Strasbourg, France). Transmonolayer electrical resistance (TER) was measured with a Millicell-ERS apparatus (Millipore, Molsheim, France). The permeability of FITC-dextran (MW 70 kDa, Invitrogen, Cergy Pontoise, France) through cell monolayers was determined in the apical-to-basolateral directions. An FITC-dextran aliquot of 0.5 mg ml^−1^ was added to the apical side of culture at the beginning of infection. The basolateral side was sampled after 30 min (initial value) and 7 h of infection. Levels of FITC-dextran in the samples were determined with a fluoroscan (FLUOstar OPTIMA) using an excitation wavelength of 485 nm and a detection emission at 538 nm. The permeability of each monolayer was normalized to the initial value determined 30 min after FITC-dextran addition.

### Adhesion and Invasion Assays

Bacterial adhesion to T84 cells was quantified in the presence of the yeast or not using the plate dilution method as previously described [18]. After 3 h of infection, bacteria and yeasts present in the culture medium were eliminated by extensive washes with sterile PBS. Cells were then trypsinized and lysed in water containing 0.1% bovine serum albumin (BSA). The cell lysates contained “cell-associated bacteria” corresponding to adherent as well as intracellular bacteria. For the determination of invasion, after PBS washes, monolayers were incubated for an additional hour with DMEM/F-12 containing 100 µg of gentamicin per ml. Since gentamicin was not concentrated in epithelial cells, intracellular bacteria survived to the incubation, while adherent and extracellular bacteria were killed. The monolayers were then washed with sterile PBS, and epithelial cells with intracellular bacteria were detached by trypsin and lysed as described elsewhere. The percentage of invasion was calculated as follows:
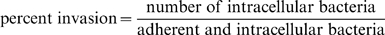



### Electron Microscopy

For transmission electron microscopy, T84 cells were fixed *in situ* with 1.6% glutaraldehyde with or without 0.075% ruthenium red in 0.1 M cacodylate buffer pH 7.5 at room temperature. Cells were washed five times in the same buffer, i.e. with or without 0.075% ruthenium red and post-fixed for 1 h at room temperature in 1% osmium tetroxyde with or without 0.075% ruthenium red in cacodylate buffer. Cells were then rinsed with distilled H_2_O, dehydrated with ethanol and embedded in Epon (Embed 812). Blocks were cut conventionally, stained and examined with a Philips CM12 microscope operating under standard conditions.

For scanning electron microscopy cells were fixed *in situ* with 1.6% glutaraldehyde in 0.1 M cacodylate buffer (pH 7.5) at room temperature. Samples were washed with the same buffer, dehydrated with increasing ethanol series, treated with hexamethyldisilazane and air dried. Cells were coated by with 3 nm of gold-palladium and observed at low voltage with a Jeol 7400F scanning electron microscope.

### Measure of Rac Protein Activation by Effector-Binding Pull-Down

HeLa cells were seeded in 100 mm Petri dishes. At 70–90% confluence, cells were depleted overnight in serum-and antibiotics-free medium supplemented with 0.1% BSA (Sigma). Infections were carried out in this medium with 3×10^8^ bacteria in the presence (or not) of 3×10^7^ yeast overnight. At indicated time cell monolayers were washed in PBS at 4°C and lysed at 4°C in cell lysis buffer (25 mM Tris-HCl pH 7.5, 150 mM NaCl, 5 mM MgCl_2_, 0.5% Triton X100, 4% glycerol, 10 mM NaF) supplemented extemporaneously with 1 mM PMSF, 2 mM Na_3_VO_4_, 2 mM DTT, 20 mM β-glycerophosphate. Lysates were centrifuged 10 min at 10,000 g at 4°C. An aliquot of 50 µl was collected (Total Rho protein input) and mixed with Laemmli blue buffer (4% SDS, 20% glycerol, 10% 2-mercaptoethanol, 0.004% bromophenol blue, 0.125 M Tris-HCL). Lysates were incubated with 30 µg of GST-PAK^70–106^ bound to glutathione-agarose beads (Sigma-Aldrich, France) 45 min at 4°C on a rotating shaker. Beads were washed twice with 1 ml of lysis buffer. Beads were mixed with 30 µl of Laemmli blue buffer, and proteins were resolved on a 12 SDS-PAGE and transferred onto PVDF transfer membrane. Immunoblots were performed using monoclonal antibodies incubated overnight at 4°C at a dilution of 1∶1000 for anti-Rac1 (clone 102; Transduction Laboratories, France) antibody, followed by a peroxidase-conjugated sheep anti-mouse (Amersham Biosciences). The presence of antibodies was revealed with the ECL detection system. The density of bands was quantified using a Multi Gauge software (Fuji Film Global).

### Recruitment of Rac Protein to Cellular Membrane

HeLa cells were seeded in 100 mm Petri dishes and infected as described above. After 3 h of infection, cell dishes were chilled on ice and rinsed twice with cold PBS. Further steps were performed at 4°C. Plates were scraped in 5 ml of cold PBS, centrifuged 5 min at 1,000 rpm, and pellets were homogenized in 0.25 ml of cold BSI buffer (3 mM imidazole, pH 7.4, 250 mM sucrose) supplemented extemporaneously with 1 mM PMSF. The mixture was transferred to a 1.5 ml tube, and cells were lysed by passing 20 times through a 1-ml syringe (U-100 Insulin, Terumo). Nuclei were removed by centrifugation 10 min at 10,000 g at 4°C. Post-nuclear supernatants (PNS) were centrifuged 1 h at 100,000 g at 4°C. Supernatants (cytosolic fractions) were transferred in a next 1.5 ml tube, and pellets (membrane fractions) were homogenized in an equal volume of BSI. Pellets and supernatants were mixed with Laemmli blue buffer and proteins were resolved on a 12% SDS-PAGE and transferred onto PVDF transfer membrane. Immunoblots were performed using monoclonal antibodies incubated overnight at 4°C at a dilution of 1∶1000 for anti-Rac1 (clone 102; Transduction Laboratories, France), 1∶3000 for anti-RhoGDI polyclonal antibodies (A-20 Santa Cruz) and 1∶2000 for anti-Human Transferrin Receptor monoclonal antibodies (ZYMED Laboratories). The density of bands was quantified with Multi Gauge software (Fuji Film Global).

### IL-8 Assay

IL-8 assays were performed on monolayers grown in six-well tissue culture plates. Cells were incubated with the yeast (overnight or not) and infected with ST. At the indicated times, the culture supernatants were centrifuged for 10 min at 10,000 rpm to pellet residual bacteria. The IL-8 concentration was determined with the Quantikine Human IL-8 Immunoassay (R&D System, Abington, U.K.), according to the manufacturer's instructions.

### Real-Time Quantitative Polymerase Chain Reaction

Briefly, after extraction of total RNA from cell culture by the acid guanidium thiocyanate-phenol-chloroform method, the RNA was converted to complementary DNA (cDNA) using oligo (dT)_18_ in 20 µL reverse-transcription reaction solution (Fermentas, France) and then used for polymerase chain reaction. Reverse transcribed cDNA were then amplified using the SYBR Green PCR Core Reagents Kit (Eurogentec, France) in special optical 96-well microtiter plates (Applied Biosystems, Courtaboeuf, France) in an ABI PRISM 7000 Sequence Detection System (Applied Biosystems), according to the manufacturer's instructions. The primers used were as follow: IL-8 sense, 5′-AAGGAACCATCTCACTGTGTGTAAAC-3′; IL-8 antisense, 5′-ATCAGGAAGGCTGCCAAGAC-3′. The reference housekeeping gene RPLP0 (encoding human acidic ribosomal phosphoprotein P0) was used for all calculations as it showed no change in expression upon treatment of cells.

### Western Blotting

At the indicated times, the infected cells were washed with PBS and scraped at 4°C in lysis buffer, solubilized for 30 min at 4°C, then centrifuged at 14,000 rpm for 20 min at 4°C, as previously described [19]. The protein concentration of the supernatant was determinated using BioRad DC reagents. Equal amounts (50 µg) of whole cell lysates were subjected to 12 SDS-polyacrylamide gel. The proteins were transferred onto a polyvinylidene fluoride membrane (PVDF Hybond-P, Amersham, Orsay, France), and incubated overnight at 4°C with anti-phospho-ERK1/2, anti-phospho-p38, anti-phospho-JNK, anti-phospho-IκB-α, anti-IκB-α rabbit antibodies (Cell Signaling Technology), or anti-ERK2, anti-p38, anti-JNK (Santa Cruz Biotechnology) and HRP-conjugated anti-rabbit antibodies (New England Biolabs). The presence of antibodies was revealed with the Enhanced Chemiluminescence detection system (ECL, Amersham).

### Electrophoretic Mobility Shift Assay (EMSA)

Cells were washed and infected with *S.* Typhimurium in the presence of the yeast (overnight or at the same time of bacterial infection) or not. At the indicated times, the infected cells were washed with cold PBS. NF-κB DNA binding activities were analyzed in total cleared cellular extracts prepared in Totex buffer (20 mM HEPES pH 7.9, 350 mM NaCl, 20% glycerol, 1% NP40, 1 mM MgCl_2_, 0.5 mM EDTA, 0.1 mM EGTA, 1 mM DTT, 1 mM PMSF, 2 µg ml^−1^ aprotinin). Samples (10 µg) were incubated for 25 min at 25°C with radiolabeled double-stranded oligonucleotide containing the κB site (5′-GATCCAAGGGGACTTTCCATG-3′). The specificity of the complexes was analyzed by incubation with an excess of unlabeled κB oligonucleotides. Complexes were separated by electrophoresis on a 6% non-denaturing polyacrylamide gel in 0.5 X TBE as previously described [19]. The dried gels were autoradiographed (Amersham).

### Statistical Analysis

All the experiments were repeated at least three times. Results are presented as the mean ± the standard error of the mean (SEM). Statistical significance was determined by analysis of variance with the StatView program for MacIntosh, followed by post hoc comparison with the Bonferroni and Dunn tests. The level of significance was set at *P*<0.05.

## Supporting Information

Figure S1 Fecal populations of *S*. Typhimurium in gnotobiotic NIH mice treated (Sb + ST) or not (ST) with *S. boulardii* for 10 days before the challenge with the bacteria. Fecal population numbers of *S. boulardii* (Sb). Arrow indicates the day of pathogenic challenge. N = 3 animals in each group.
(2.51 MB TIF)
