# Correction: Interaction of *Saccharomyces boulardii* with *Salmonella enterica* Serovar Typhimurium Protects Mice and Modifies T84 Cell Response to the Infection

**DOI:** 10.1371/journal.pone.0267067

**Published:** 2022-04-11

**Authors:** Flaviano S. Martins, Guillaume Dalmasso, Rosa M. E. Arantes, Anne Doye, Emmanuel Lemichez, Patricia Lagadec, Veronique Imbert, Jean-François Peyron, Patrick Rampal, Jacques R. Nicoli, Dorota Czerucka

Concerns have been raised about results reported in Figs 7, 9, and 10 of this article [1]:

- In Fig 7B, C, the control lanes are separated from the experimental lanes and it is unclear from the figure whether the results in each panel were obtained using a single blot or multiple blots. There also appear to be horizontal discontinuities above the hTR bands in Fig 7C. The original blot data underlying these results are in S1 File. The data indicated that for each experiment, the control lanes were included on the same blot as the experimental lanes, and data between the control and experimental lanes shown were omitted from the figure. For Fig 7B, the Total and Rac-GTP data were obtained using separate blots. For Fig 7C, the Rac, RhoGDI, and hTR data were obtained using different blots.
- There are similarities in Fig 9A between the Total p38 data and the lower bands in the Total JNK panel, when the Total JNK panel is stretched and enhanced. A higher resolution version of the results reported in Fig 9A (S2 File), confirmed that these results are more similar than would be expected for different western blots.
- The western blot panel shown in Fig 10A does not include control data to demonstrate relative protein loading across lanes or to show total IkB levels.
- For western blot panels in Figs 9 and 10, the brightness and contrast are adjusted such that background details are not visible and image integrity cannot be verified.

The original uncropped western blot images for Figs 9 and 10A are no longer available. The quantitative data for Fig 9A are in S3 File, and the original image supporting the EMSA result in Fig 10A is in S4 File.

Replicate data for the experiments reported in Figs 9 and 10 are provided in S5–S7 Files. Based on these results, the following sentences of the Results text are hereby revised:

Published:

Activation of p38 and ERK1/2 were detectable after 1 h of ST infection and increased after 2 and 3 h. Activation of JNK was not detectable until 2 h of infection. When Sb was simultaneously added with bacteria the level of phosphorylation of the three MAPKs was similar to the levels measured in cells infected by ST-alone. In contrast, pre-incubation of cells with Sb prior to ST infection decreased activation of ERK1/2 and JNK kinases (Fig. 9A). The effect of Sb on p38 activation appeared less pronounced although no more activation could be detected at 3 hs.

. . .

In T84 cells, ST induced phosphorylation of IκB-α that occurred 1 h after the beginning of infection, increased at 2 h and remained elevated over the course of 3 h (Fig. 10A). ST-induced phosphorylation of IκB-α decreased when cells were pre-incubated overnight with Sb but not when Sb and bacteria were simultaneously applied.

Revision:

Activation of all three MAPKSs (p38, JNK, ERK) was detectable by 1h of ST infection. p38 and ERK phosphorylation increased between 1h and 3h, whereas no pronounced differences were observed in phosphorylated JNK during this timeframe. When Sb was simultaneously added with bacteria the level of phosphorylation of the three MAPKs was similar to the levels measured in cells infected by ST-alone. In contrast, pre-incubation of cells with Sb prior to ST infection decreased activation of the MAPKs. This decrease was observed at all three timepoints (1h, 2h, 3h) for ERK1/2 and JNK kinases, and at the 2h and 3h timepoints for p38 (Fig. 9A).

. . .

In T84 cells, ST induced phosphorylation of IκB-α that occurred within 1 h after the beginning of infection, and remained elevated over the course of 3 h (Fig. 10A). ST-induced phosphorylation of IκB-α appeared to be slightly decreased when cells were pre-incubated overnight with Sb, but further experiments would be needed to verify this result. The phosphorylation levels did not appear to be affected when Sb and bacteria were simultaneously applied.

## Supporting information

S1 File Original data underlying Fig 7. (ZIP)

S2 File Higher resolution version of Fig 9A. (PPT)

S3 File Quantitative data to support results reported Fig 9. (ZIP)

S4 File Original data underlying EMSA results in Fig 10A. (TIF)

S5 File Replicate data for Fig 9A. (ZIP)

S6 File Replicate data for Fig 9B. (ZIP)

S7 File Replicate data for Fig 10A. (ZIP)
