# Supplementary figures and images for: Interaction of Saccharomyces boulardii with Salmonella enterica Serovar Typhimurium Protects Mice and Modifies T84 Cell Response to the Infection
Source: PLoS One. 2010 Jan 27;5(1):e8925. doi: 10.1371/journal.pone.0008925 (PMC2811747; doi:10.1371/journal.pone.0008925)

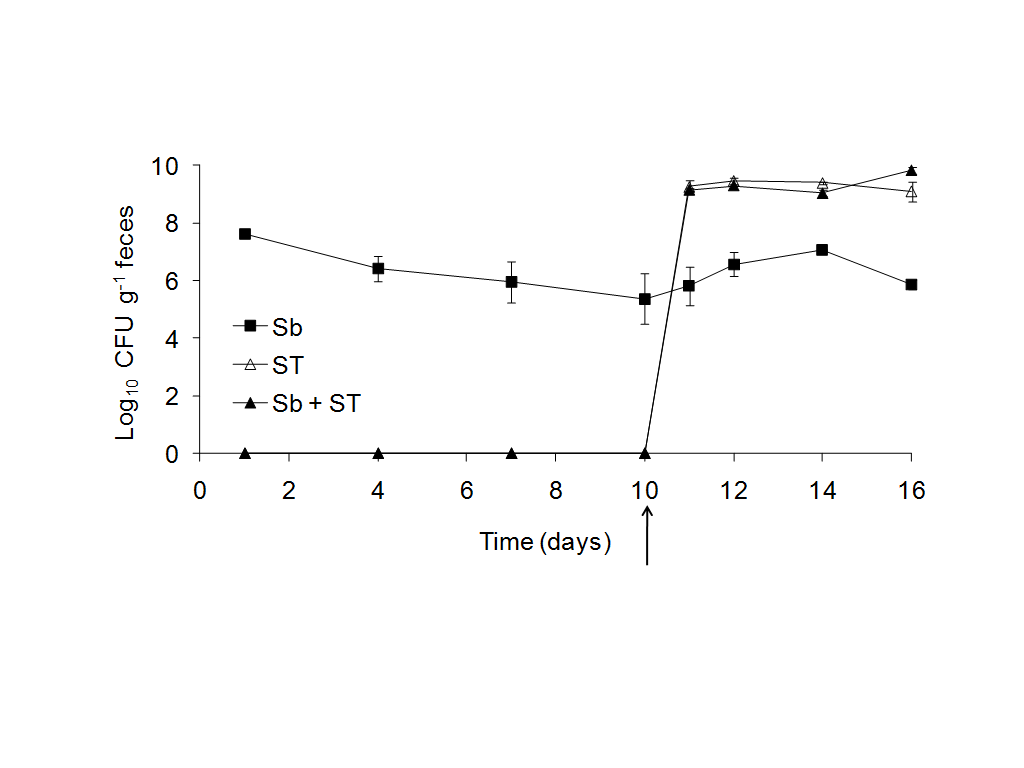

Supplement: Figure S1 — Fecal populations of S. Typhimurium in gnotobiotic NIH mice treated (Sb + ST) or not (ST) with S. boulardii for 10 days before the challenge with the bacteria. Fecal population numbers of S. boulardii (Sb). Arrow indicates the day of pathogenic challenge. N = 3 animals in each group. (2.51 MB TIF) [file pone.0008925.s001.tif]
