# Supplementary figures and images for: Correction: Interaction of Saccharomyces boulardii with Salmonella enterica Serovar Typhimurium Protects Mice and Modifies T84 Cell Response to the Infection
Source: PLoS One. 2022 Apr 11;17(4):e0267067. doi: 10.1371/journal.pone.0267067 (PMC9000030; doi:10.1371/journal.pone.0267067)

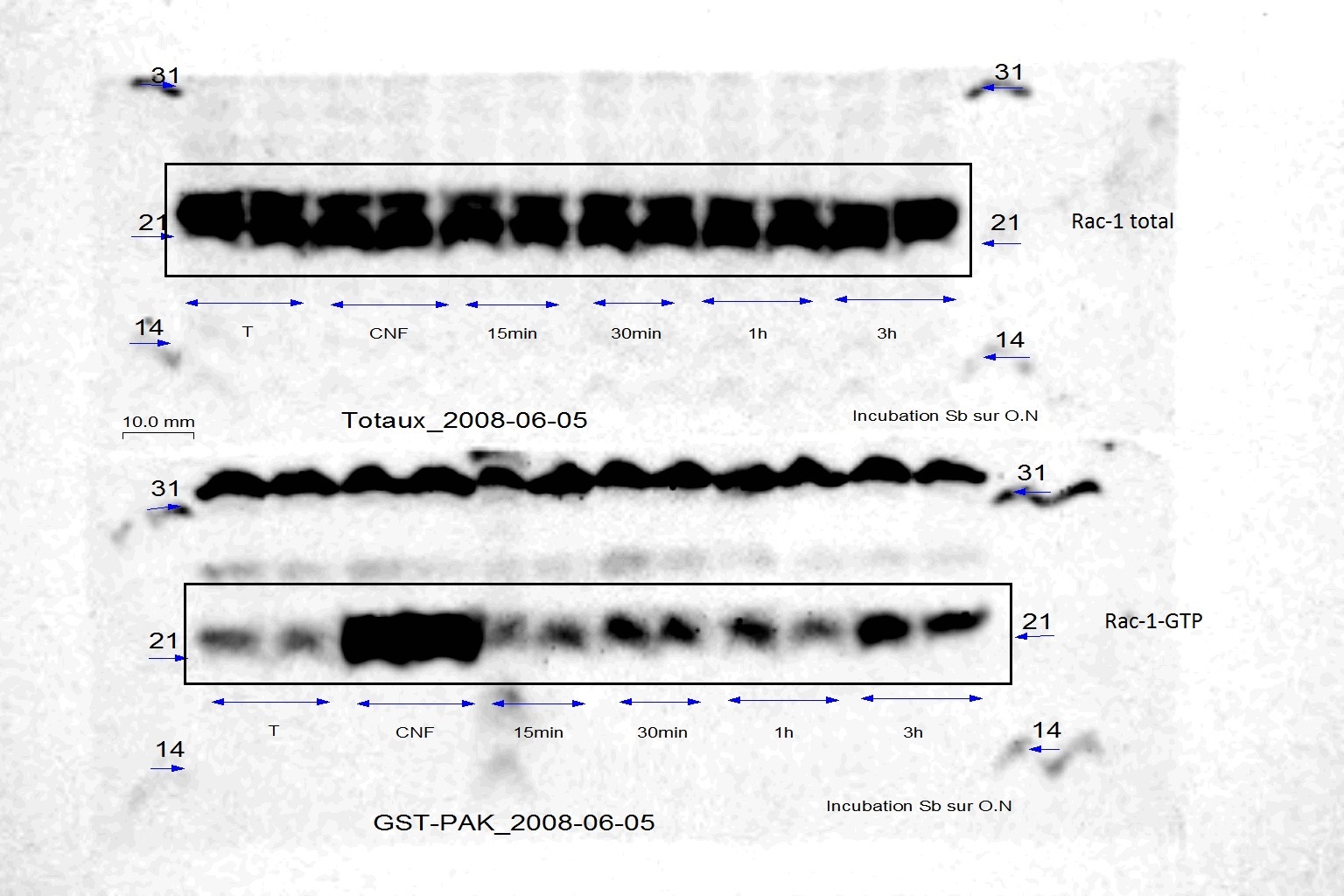

Supplement: S1 File — (ZIP) [file pone.0267067.s001.zip › Fig 7A/20080605_1444 bis.jpg]

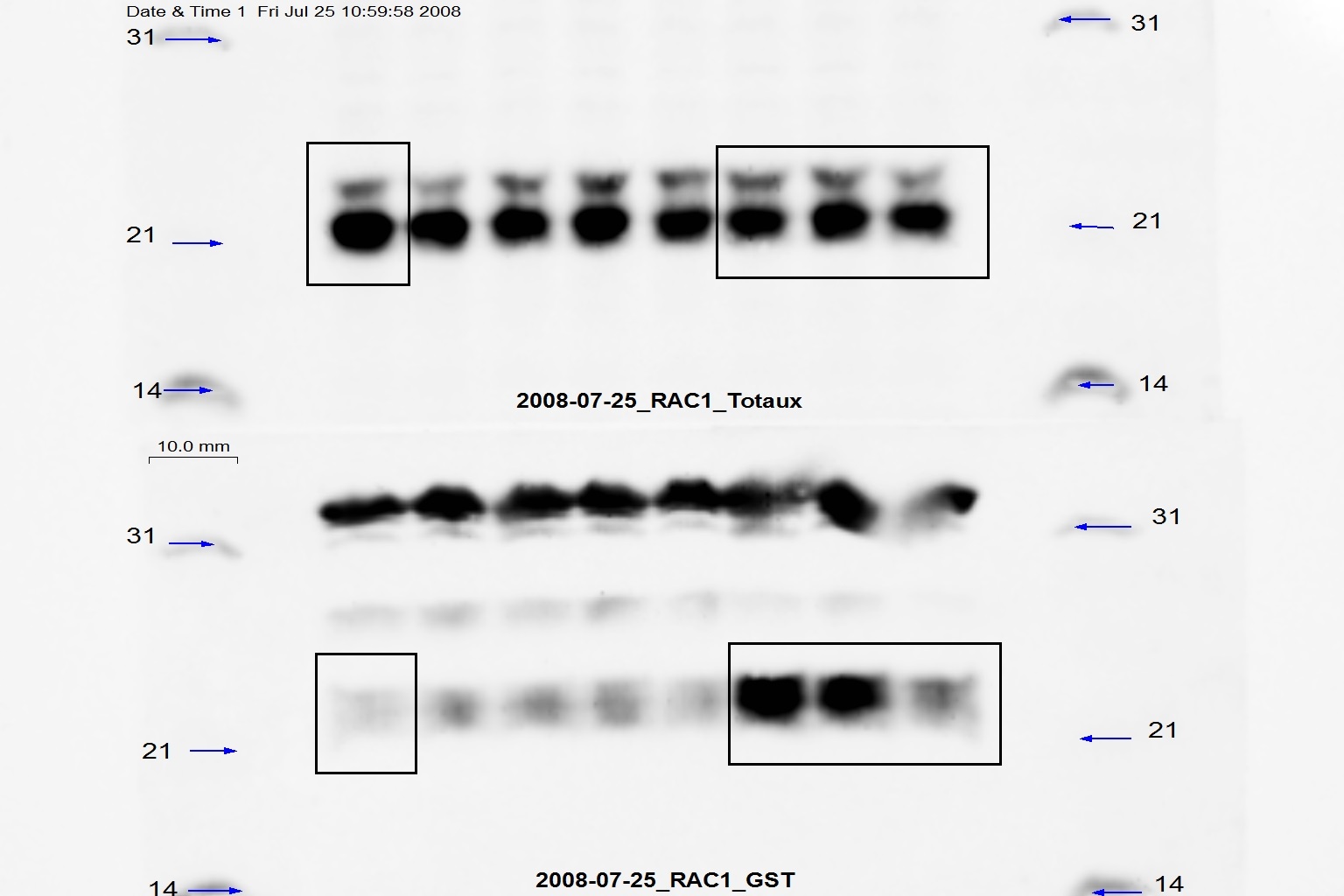

Supplement: S1 File — (ZIP) [file pone.0267067.s001.zip › Fig 7B/20080725_1123 bis.jpg]

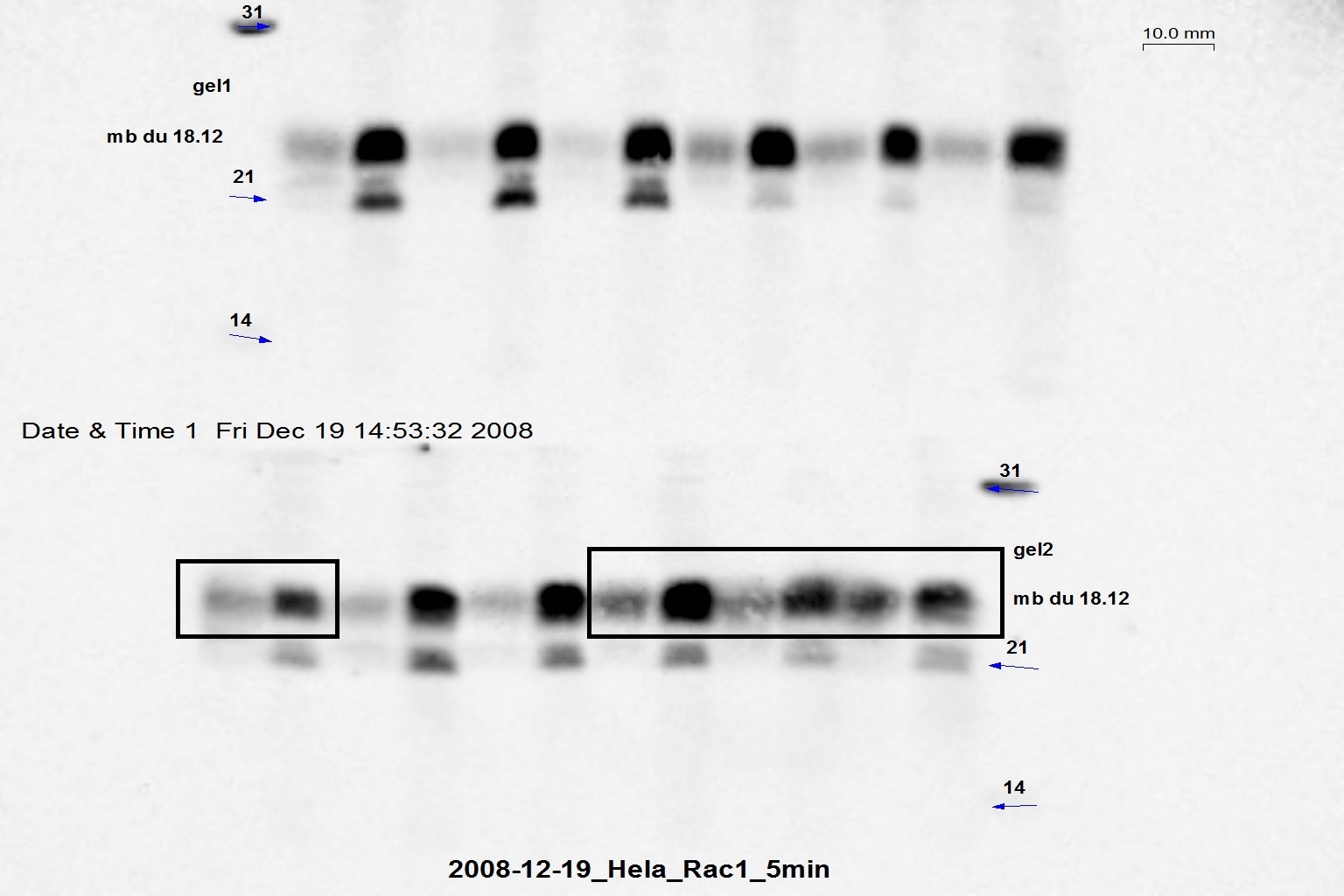

Supplement: S1 File — (ZIP) [file pone.0267067.s001.zip › Fig 7C/20081219_1500_5min bis.jpg]

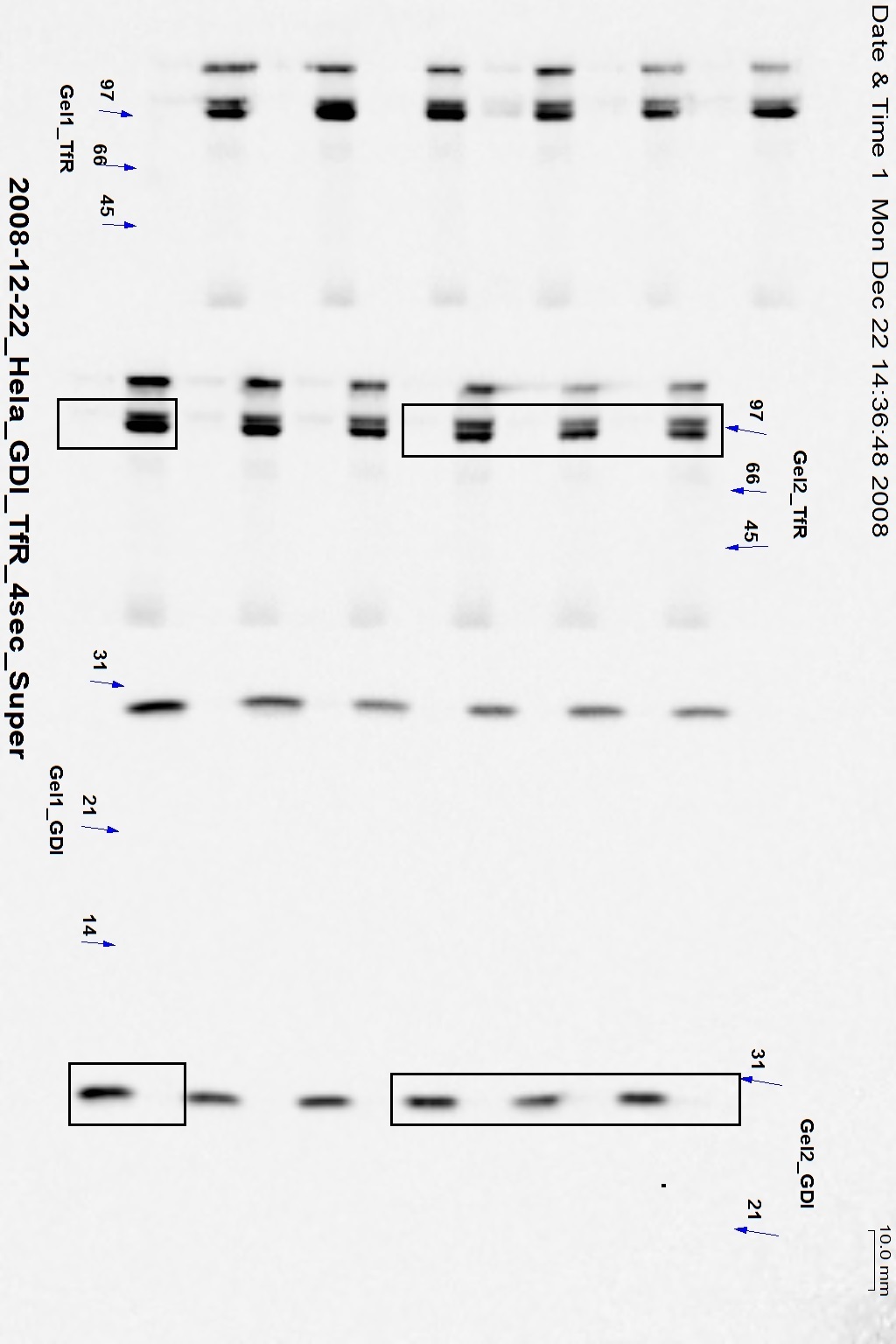

Supplement: S1 File — (ZIP) [file pone.0267067.s001.zip › Fig 7C/20081222_1437_4 sec bis.jpg]

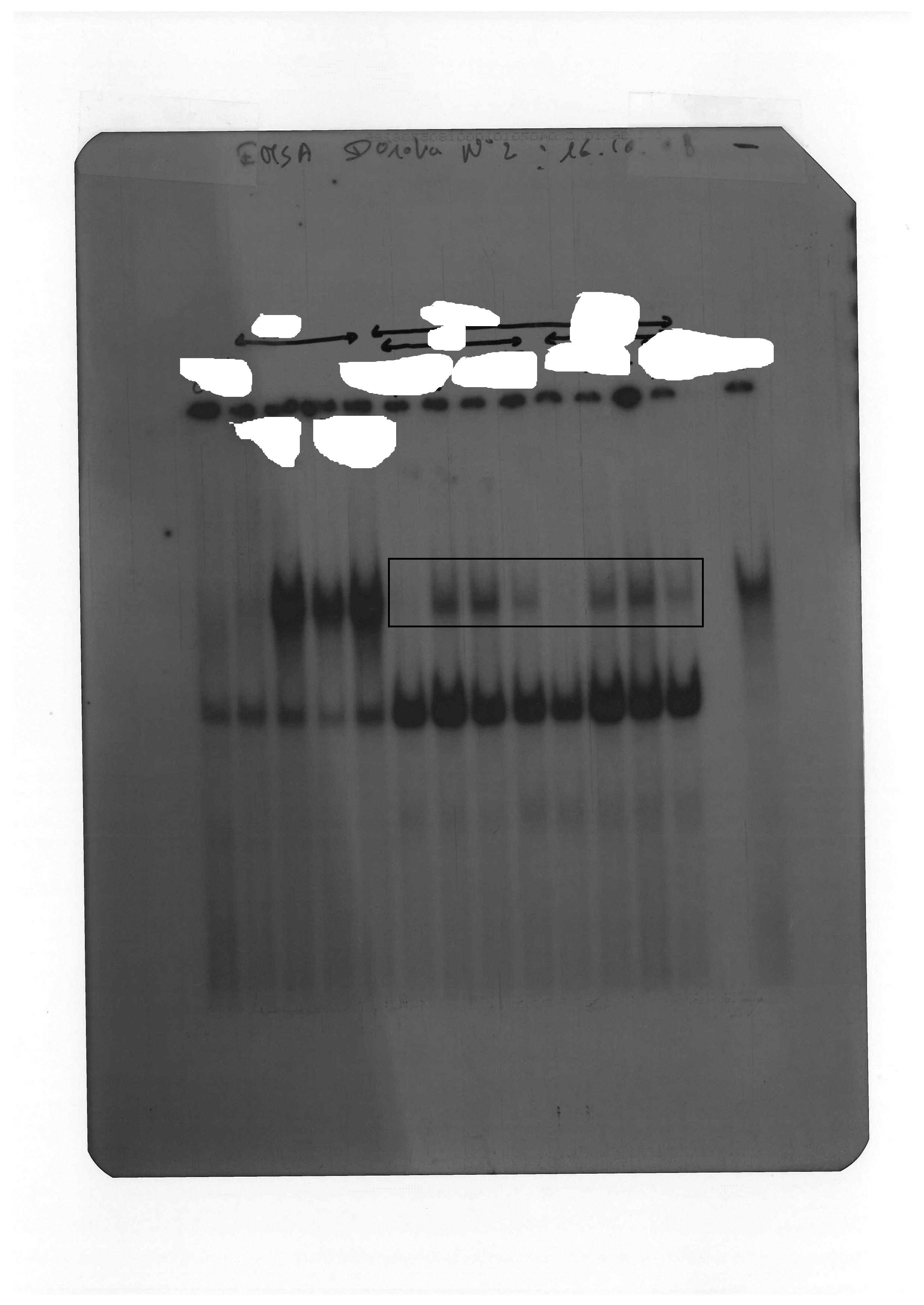

Supplement: S4 File — (TIF) [file pone.0267067.s004.tif]

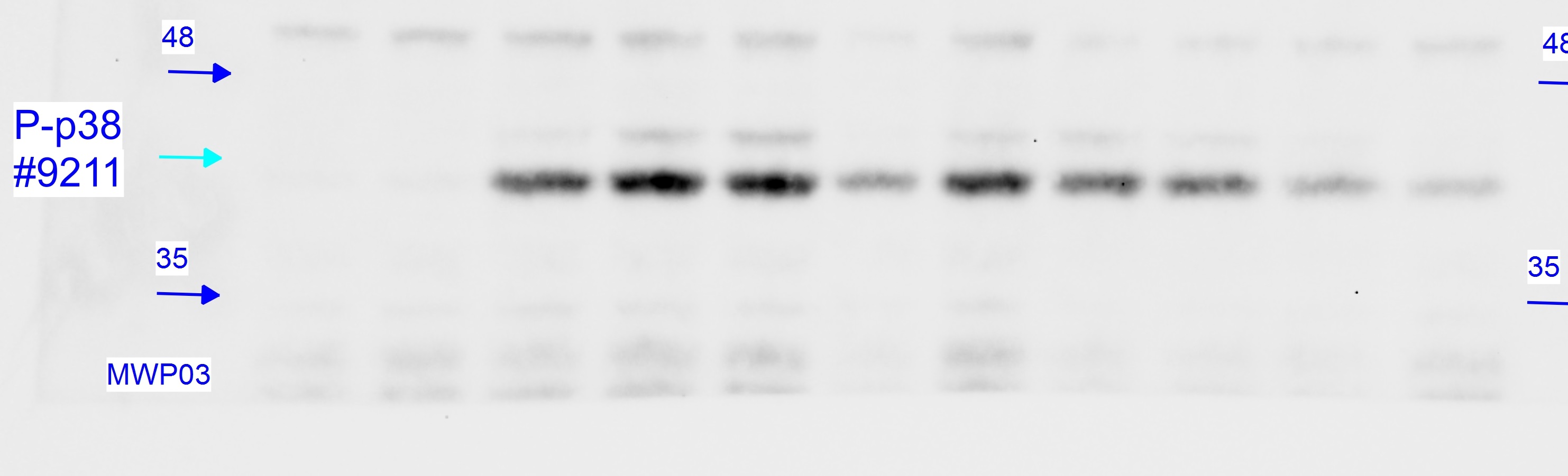

Supplement: S5 File — (ZIP) [file pone.0267067.s005.zip › mb 1_P-p38_p38-tot Fig 9A/2019-12-04 Chemi 30.000s_P-p38_crop.jpg]

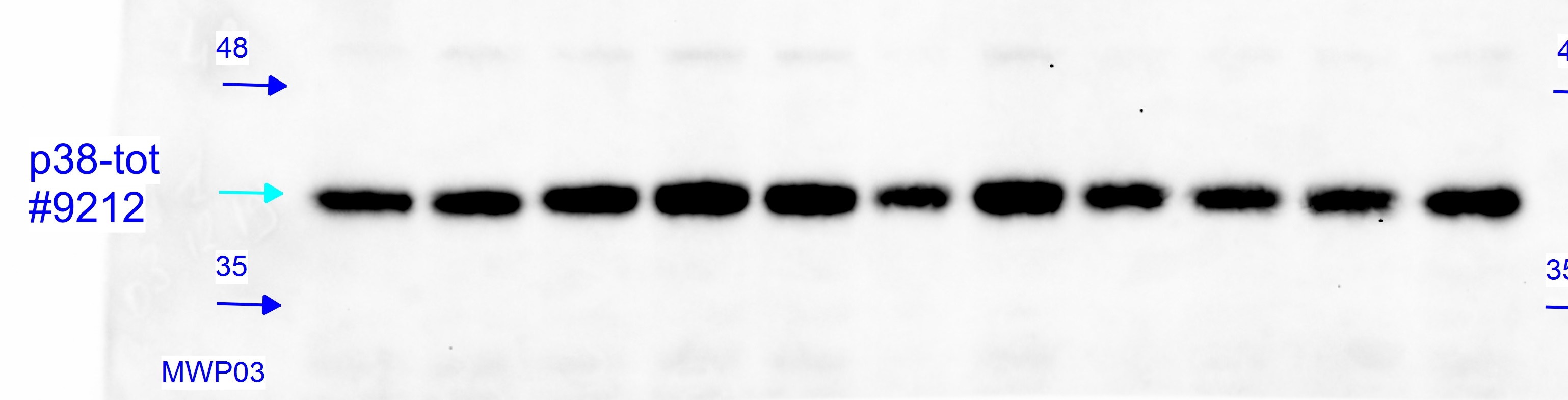

Supplement: S5 File — (ZIP) [file pone.0267067.s005.zip › mb 1_P-p38_p38-tot Fig 9A/2019-12-11 Chemi 90.000s_p38-tot_crop.jpg]

Mb #1

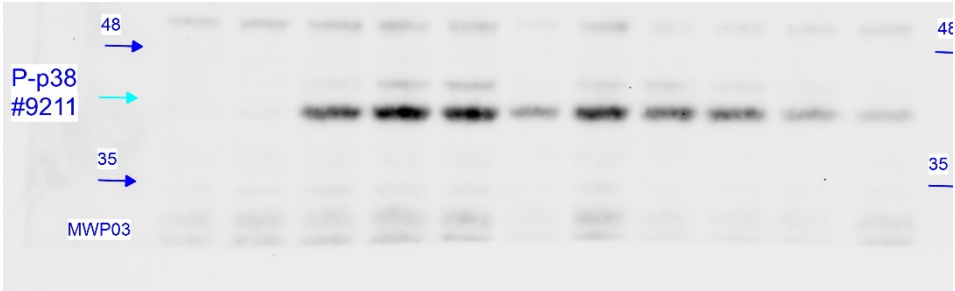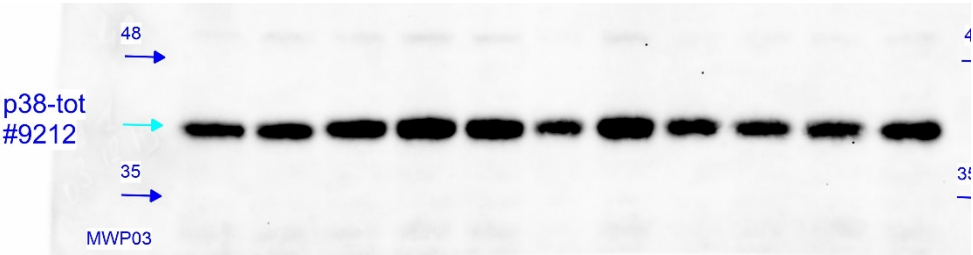

Supplement: S5 File — (ZIP) [file pone.0267067.s005.zip › mb 1_P-p38_p38-tot Fig 9A/Mb1 p38.pdf]

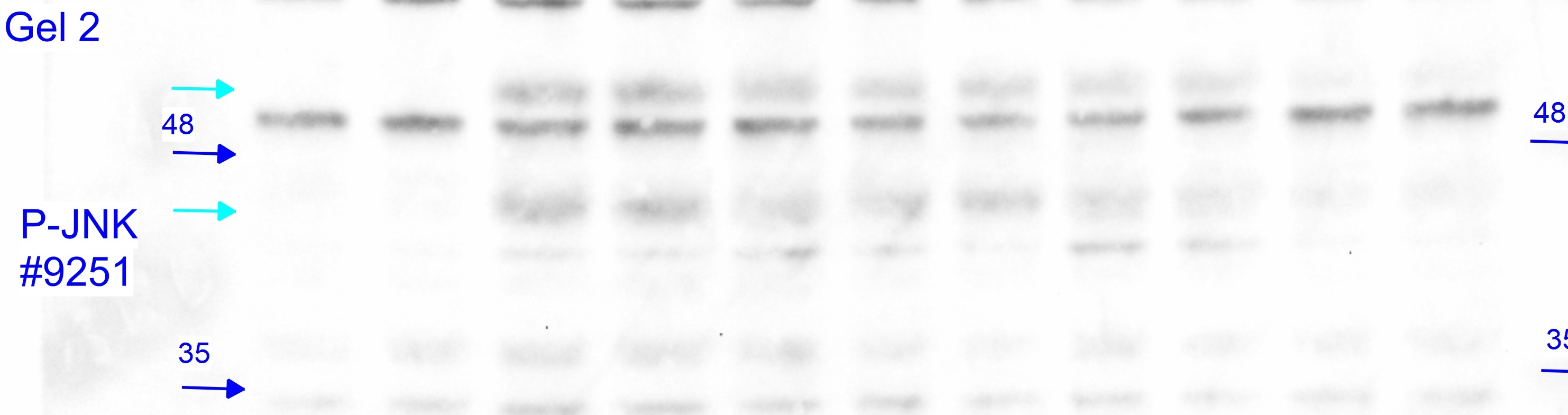

Supplement: S5 File — (ZIP) [file pone.0267067.s005.zip › mb 2_P-JNK_JNK-tot Fig 9A/2019-12-04 Chemi 60.000s_P-JNK_crop.jpg]

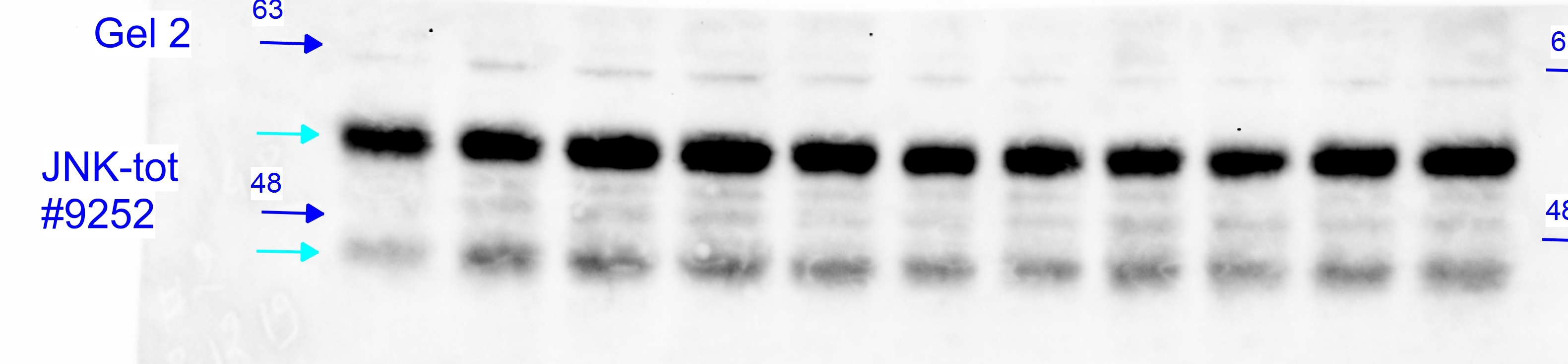

Supplement: S5 File — (ZIP) [file pone.0267067.s005.zip › mb 2_P-JNK_JNK-tot Fig 9A/2019-12-11 Chemi 90.000s_JNK-tot_crop.jpg]

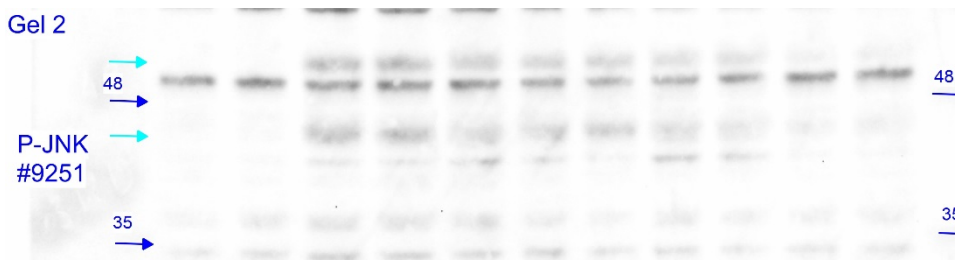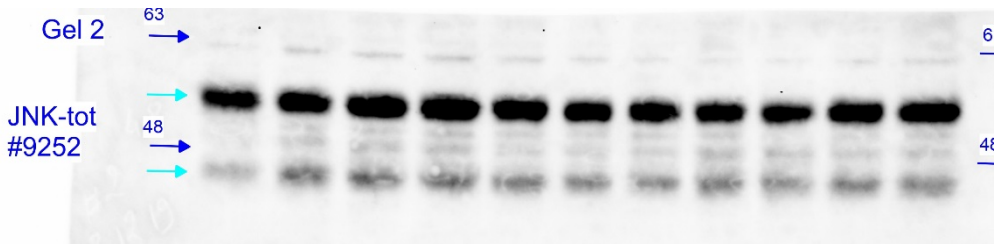

Supplement: S5 File — (ZIP) [file pone.0267067.s005.zip › mb 2_P-JNK_JNK-tot Fig 9A/Mb2 JNK.pdf]

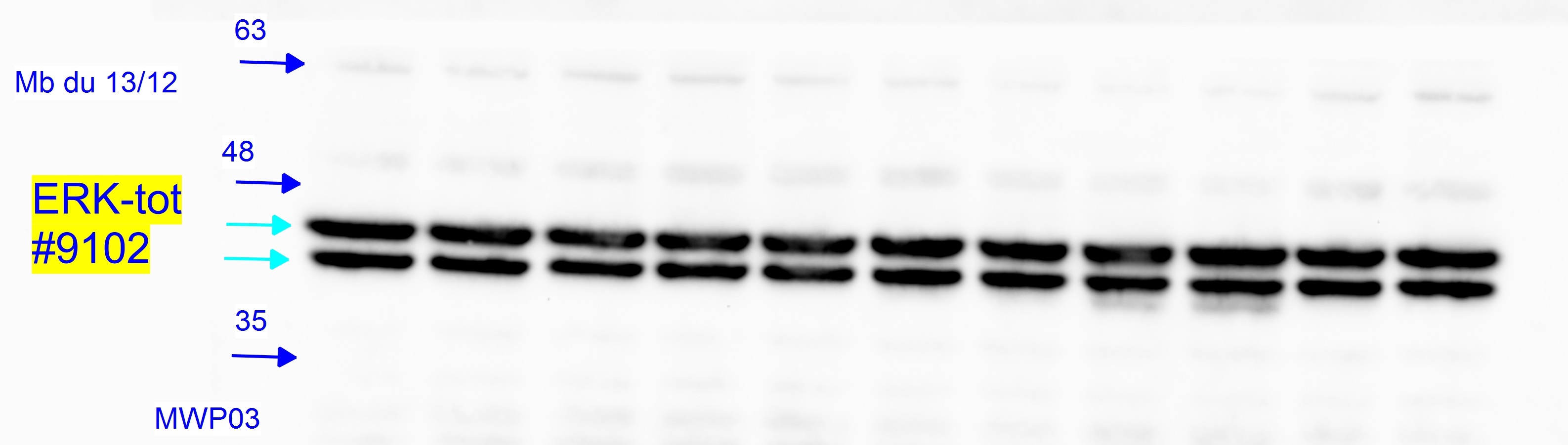

Supplement: S5 File — (ZIP) [file pone.0267067.s005.zip › mbs 4 et 5_ERK-tot_P-ERK_Fig 9A and b-actin for Fig 9A and 10A/2019-12-16 Chemi 60.000s_ERK-tot_crop.jpg]

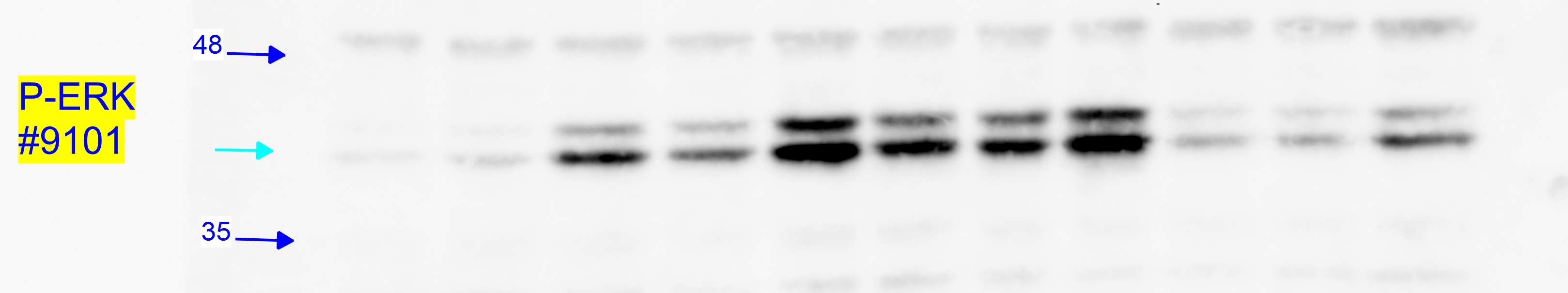

Supplement: S5 File — (ZIP) [file pone.0267067.s005.zip › mbs 4 et 5_ERK-tot_P-ERK_Fig 9A and b-actin for Fig 9A and 10A/2019-12-18 Chemi 60.000s_P-ERK_crop.jpg]

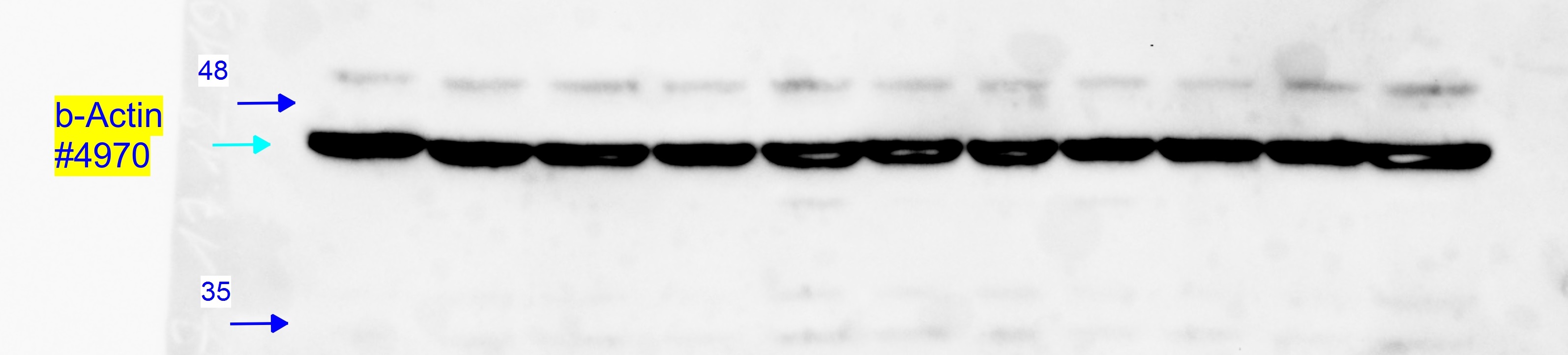

Supplement: S5 File — (ZIP) [file pone.0267067.s005.zip › mbs 4 et 5_ERK-tot_P-ERK_Fig 9A and b-actin for Fig 9A and 10A/2019-12-19 Chemi 20.000s_b-Actin_crop.jpg]

Mb#4

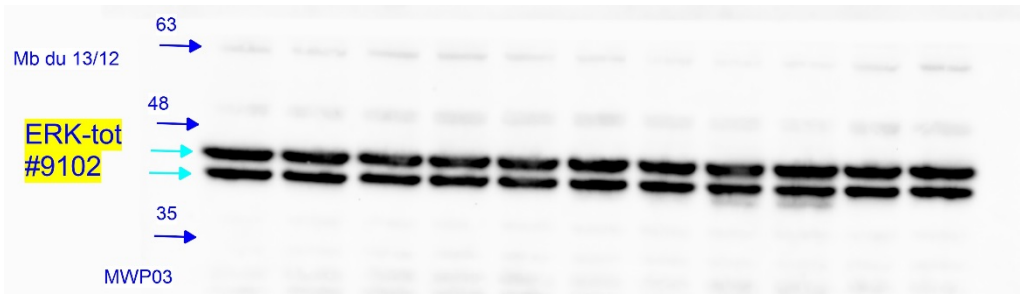

Impossible de bien dé-hybrider ERK-tot => new mb = mb#5

Mb#5

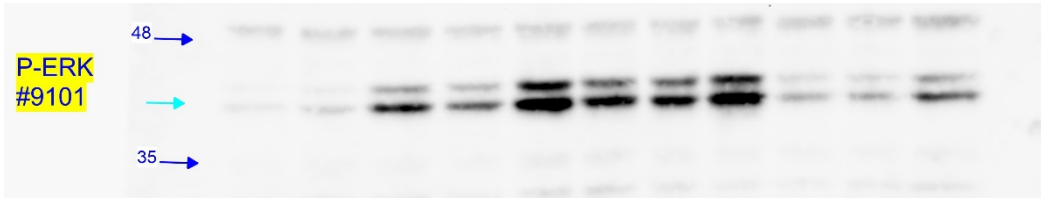

Mb#5

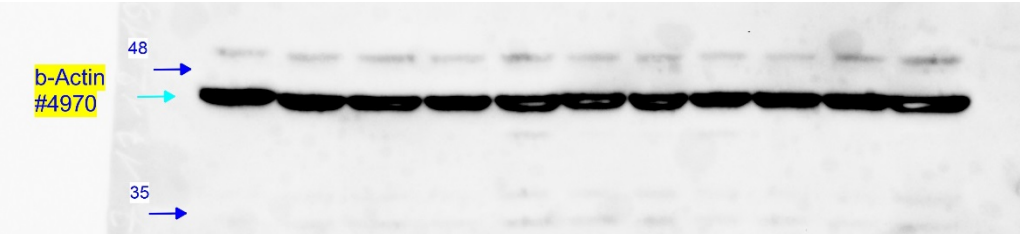

Supplement: S5 File — (ZIP) [file pone.0267067.s005.zip › mbs 4 et 5_ERK-tot_P-ERK_Fig 9A and b-actin for Fig 9A and 10A/Mbs 4&5 ERK.pdf]

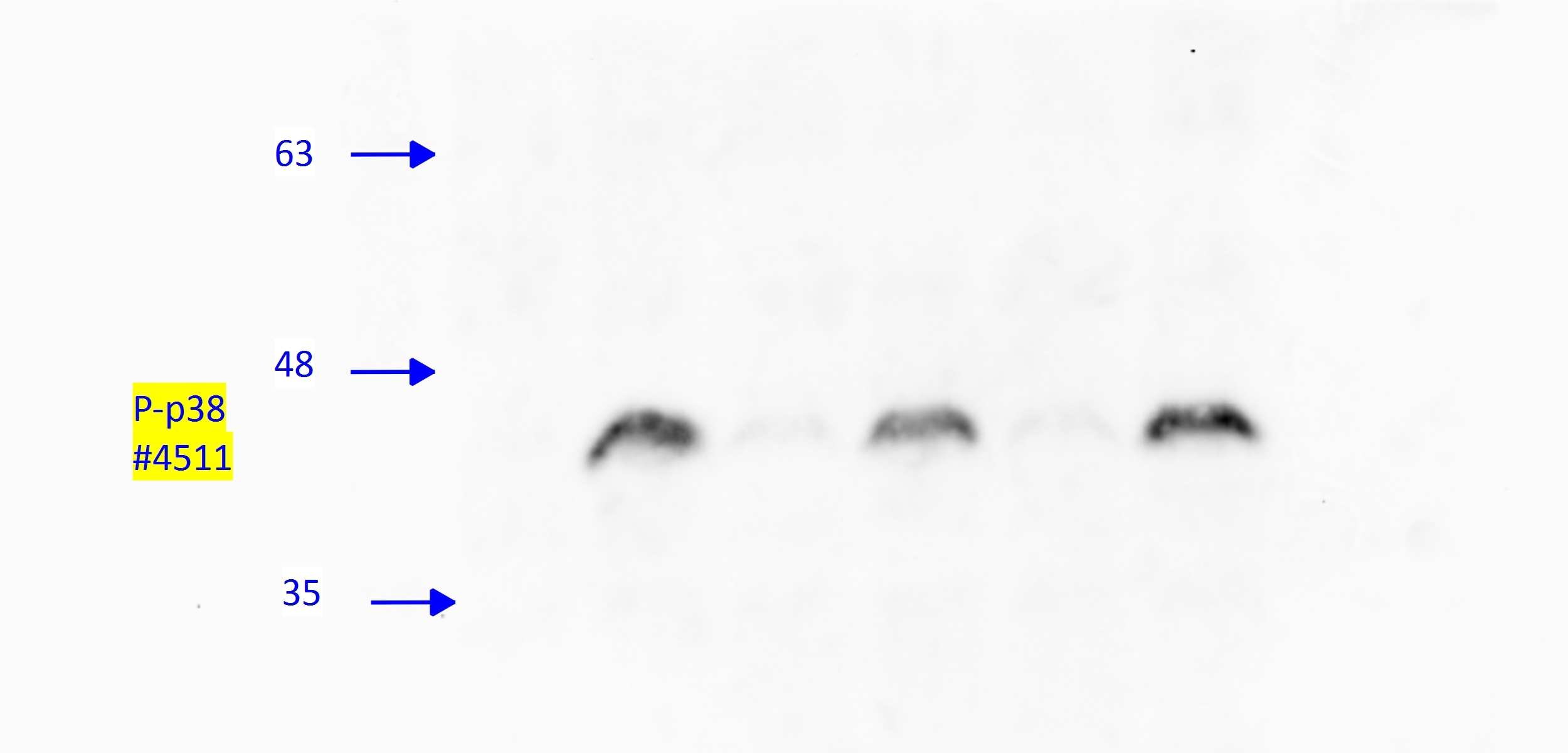

Supplement: S6 File — (ZIP) [file pone.0267067.s006.zip › 2020-01-08 Chemi 120.000s_P-p38_crop.jpg]

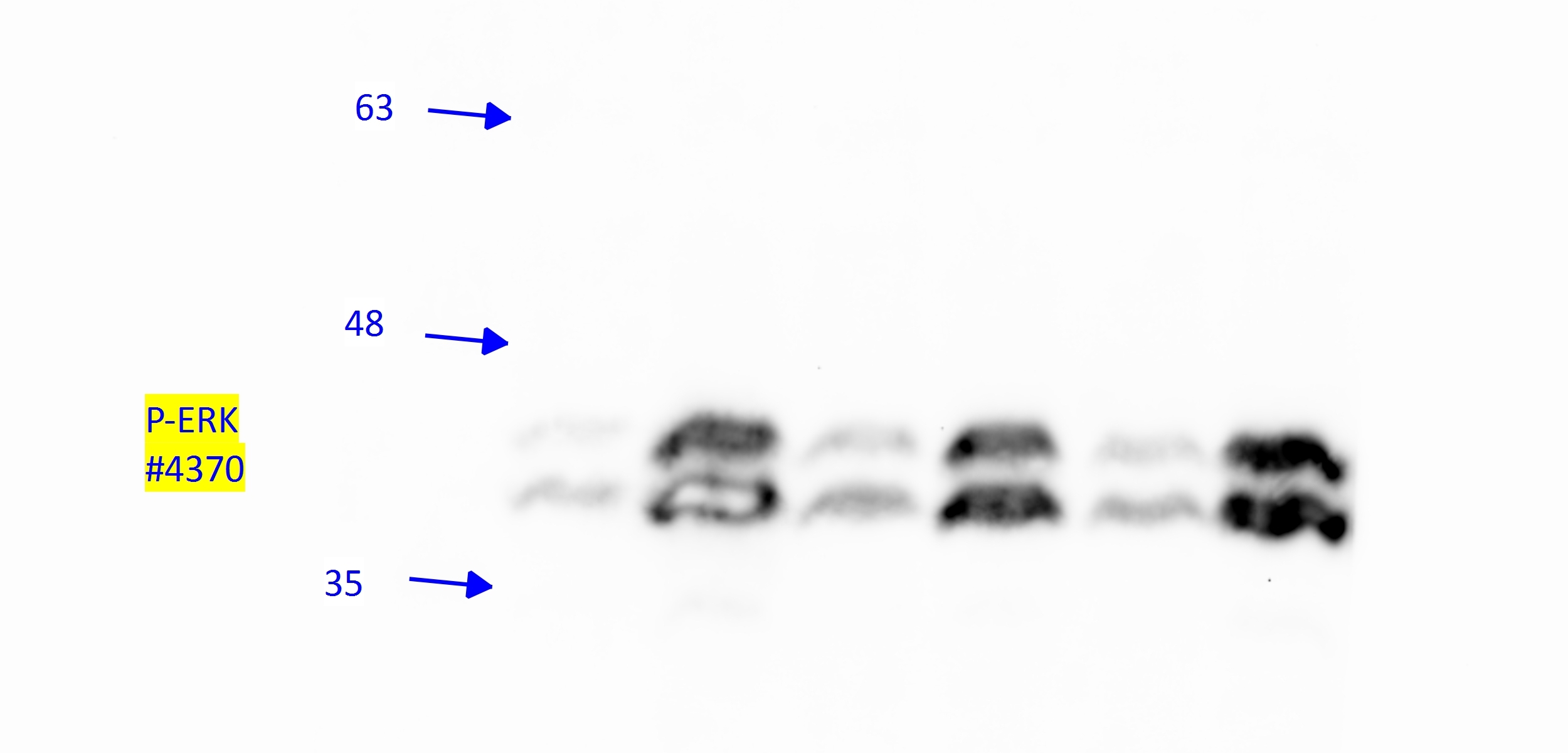

Supplement: S6 File — (ZIP) [file pone.0267067.s006.zip › 2020-01-08 Chemi 40.000s_P-ERK_2_crop.jpg]

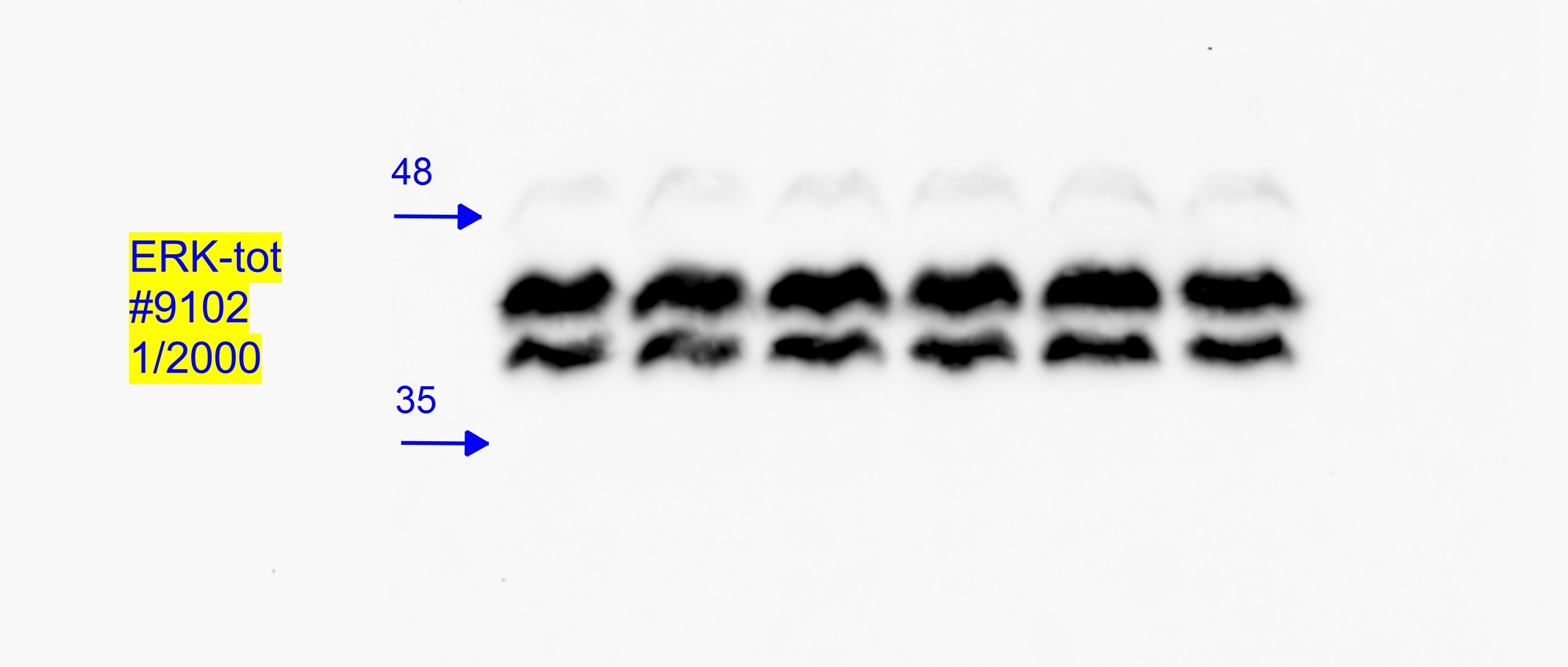

Supplement: S6 File — (ZIP) [file pone.0267067.s006.zip › 2020-01-10 Chemi 60.000s_ERK-tot_crop.jpg]

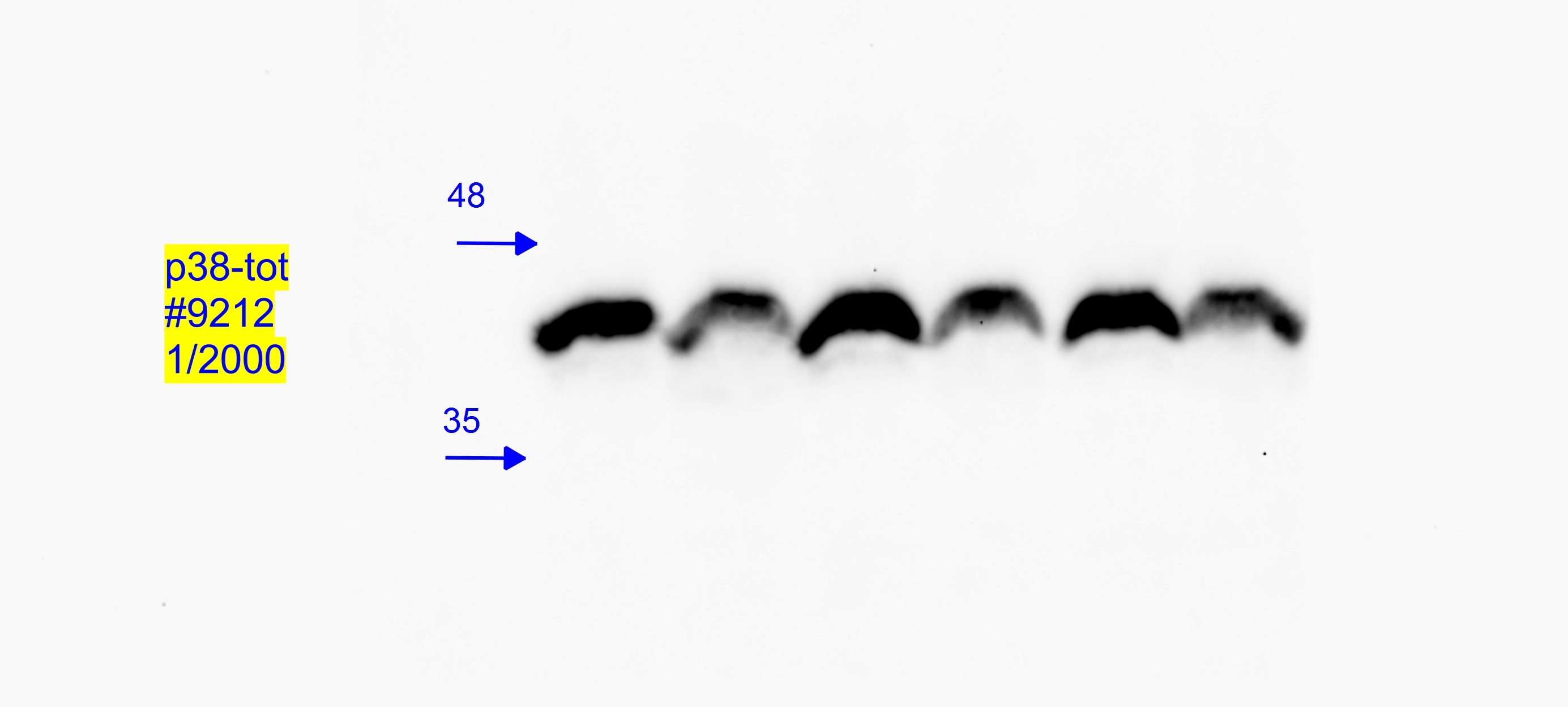

Supplement: S6 File — (ZIP) [file pone.0267067.s006.zip › 2020-01-10 Chemi 60.000s_p38-tot_crop.jpg]

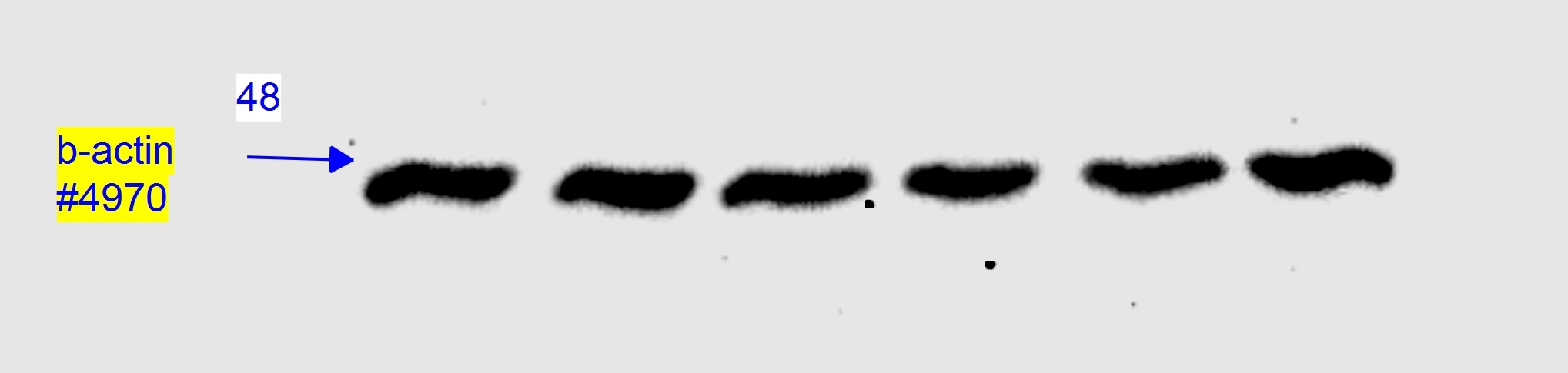

Supplement: S6 File — (ZIP) [file pone.0267067.s006.zip › 2020-01-14 Chemi 240.000s_Mb6_b-actin_crop.jpg]

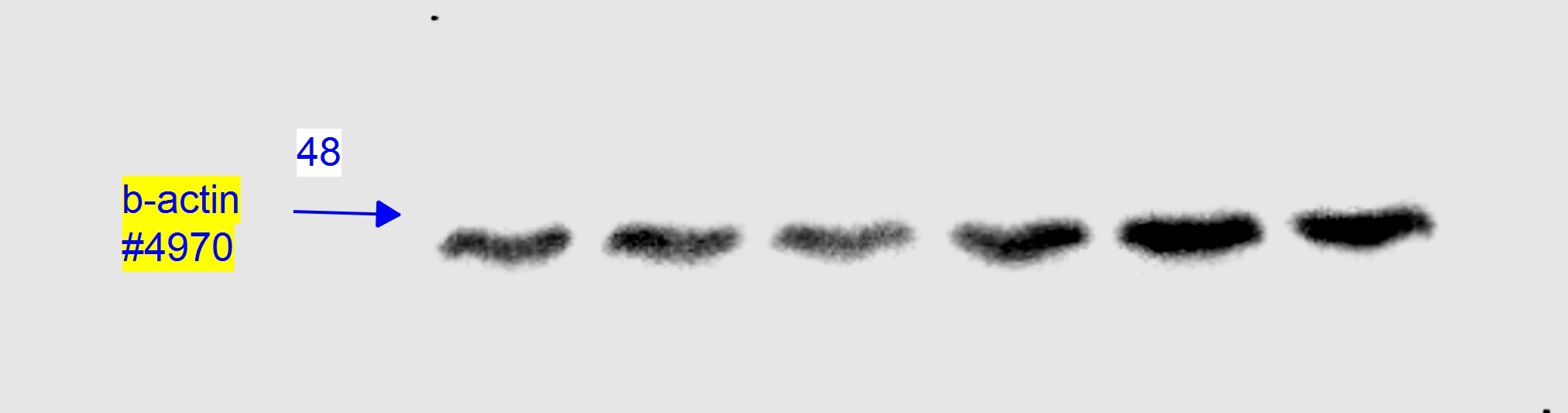

Supplement: S6 File — (ZIP) [file pone.0267067.s006.zip › 2020-01-14 Chemi 240.000s_Mb7_b-actin_crop.jpg]

Mb #6

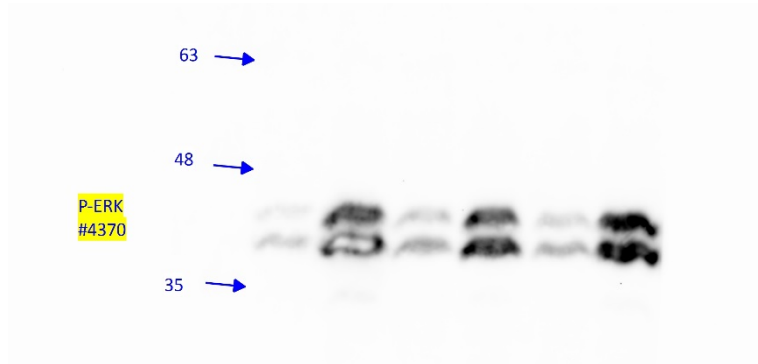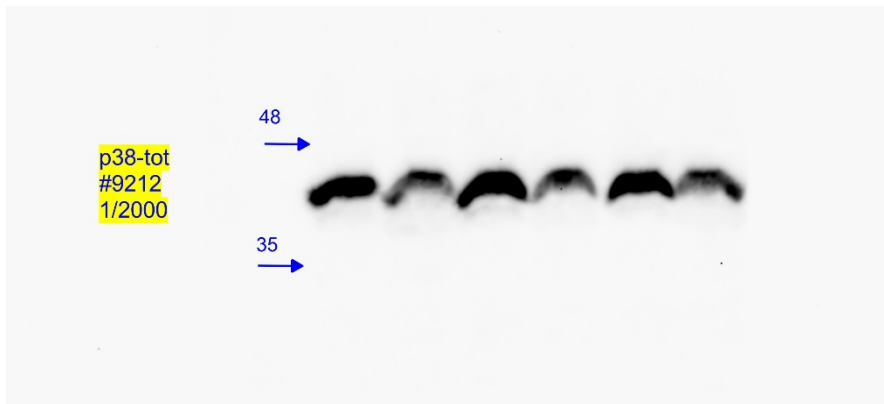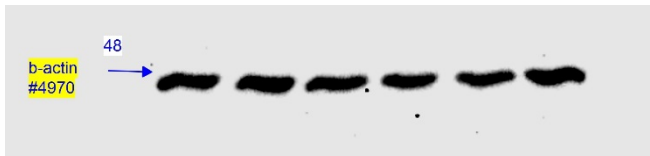

Mb #7

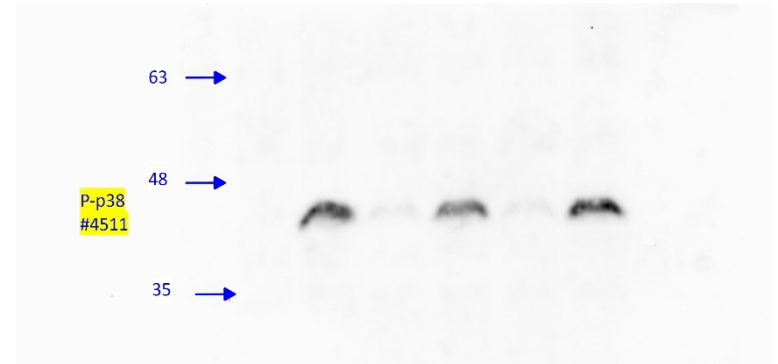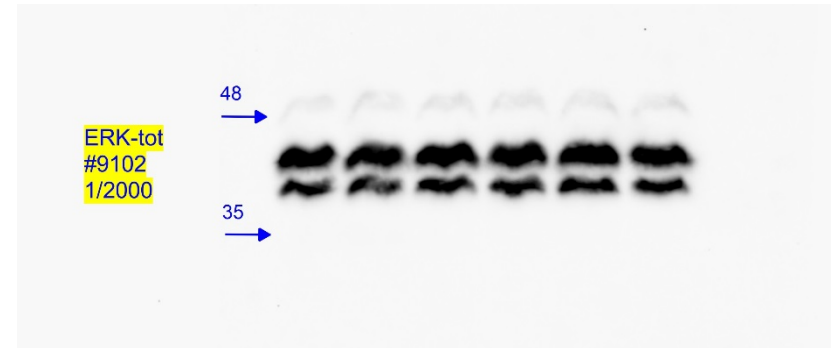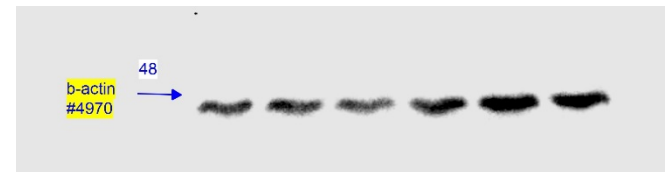

Supplement: S6 File — (ZIP) [file pone.0267067.s006.zip › Mbs 6&7.pdf]

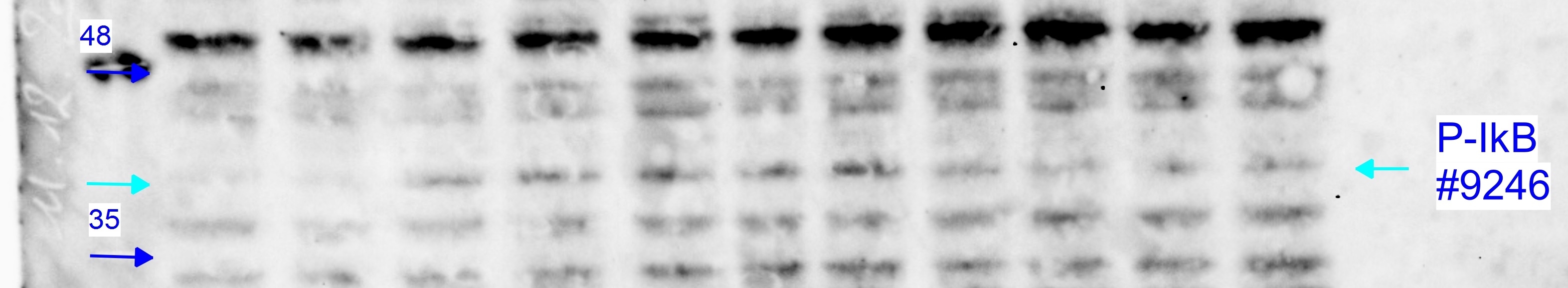

Supplement: S7 File — (ZIP) [file pone.0267067.s007.zip › 2019-12-12 Chemi 180.000s_P-IkB_crop.jpg]

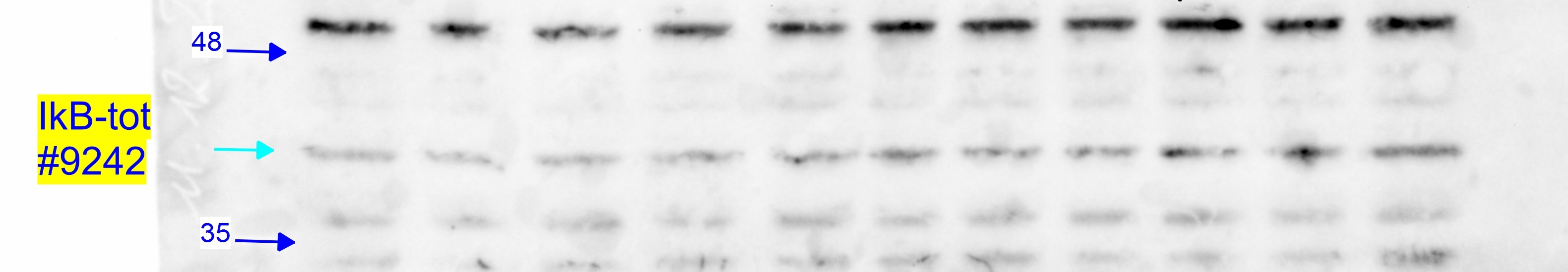

Supplement: S7 File — (ZIP) [file pone.0267067.s007.zip › 2019-12-16 Chemi 150.000s_IkB-tot_crop.jpg]

Mb #3

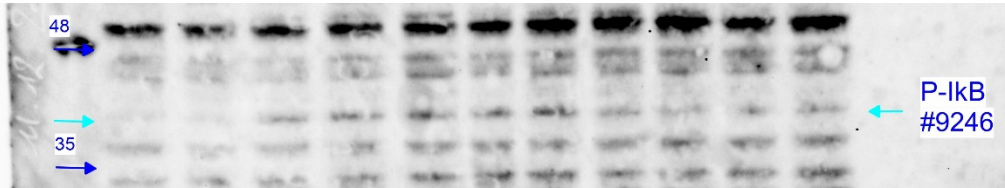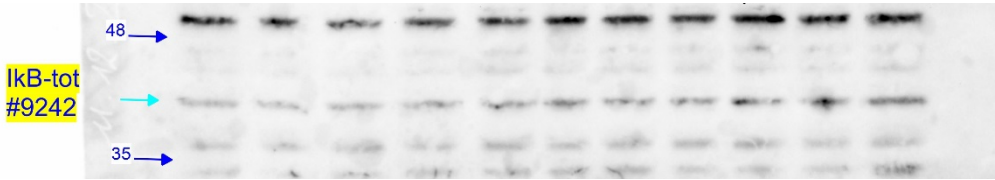

Supplement: S7 File — (ZIP) [file pone.0267067.s007.zip › Mb3 IkB.pdf]
