# Supplementary material for: Correction: Interaction of Saccharomyces boulardii with Salmonella enterica Serovar Typhimurium Protects Mice and Modifies T84 Cell Response to the Infection
Source: PLoS One. 2022 Apr 11;17(4):e0267067. doi: 10.1371/journal.pone.0267067 (PMC9000030; doi:10.1371/journal.pone.0267067)
Supplement: S2 File — (PPT) [file pone.0267067.s002.ppt]

## Slide 1
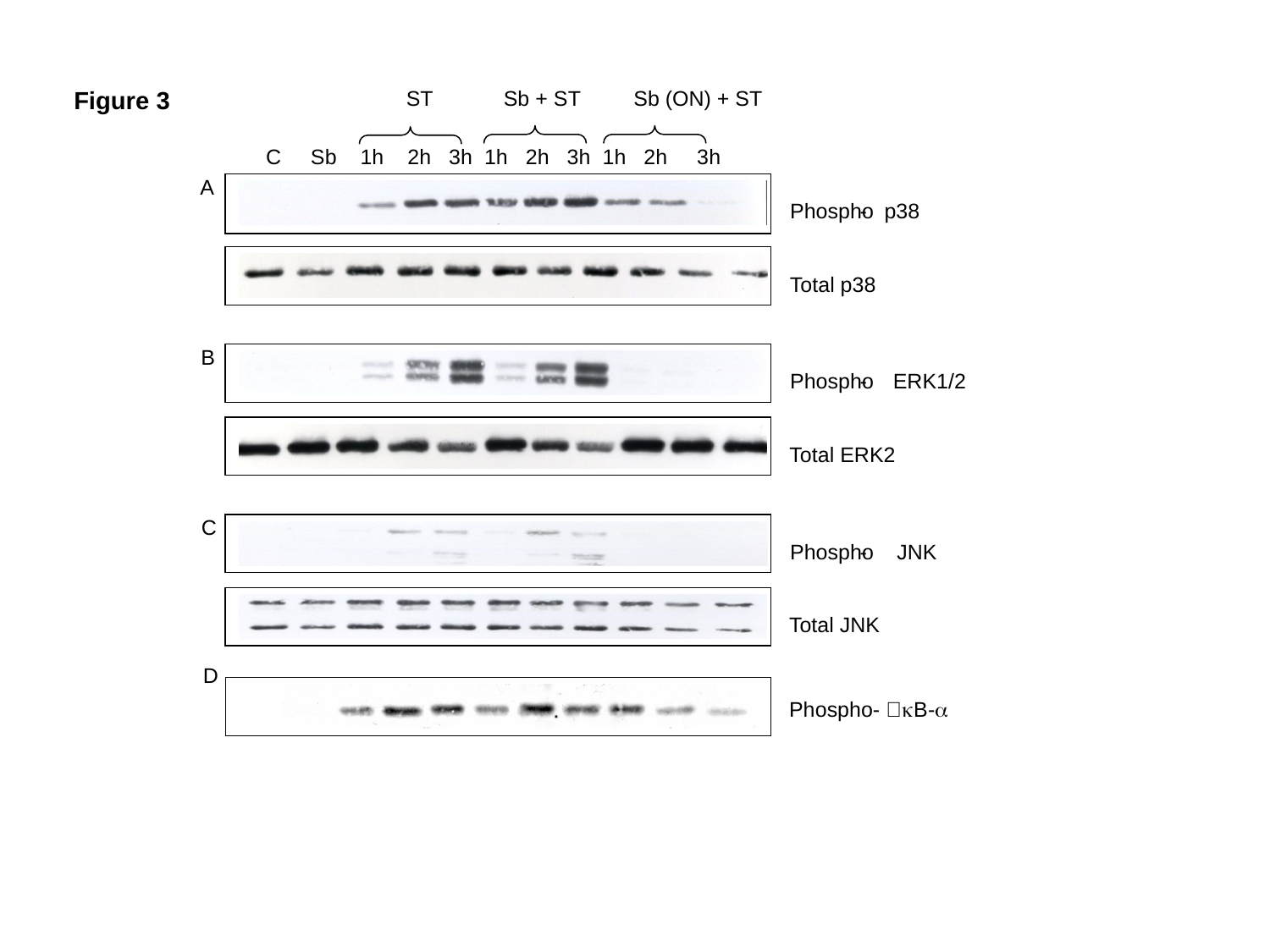

Figure 3
 ST Sb + ST Sb (ON) + ST
 C Sb 1h 2h 3h 1h 2h 3h 1h 2h 3h
A
 Phospho
-
p38
 Total p38
B
 Phospho
-
ERK1/2
 Total ERK2
C
 Phospho
-
JNK
 Total JNK
D
Phospho- B-α
