# Supplementary material for: Correction: Interaction of Saccharomyces boulardii with Salmonella enterica Serovar Typhimurium Protects Mice and Modifies T84 Cell Response to the Infection
Source: PLoS One. 2022 Apr 11;17(4):e0267067. doi: 10.1371/journal.pone.0267067 (PMC9000030; doi:10.1371/journal.pone.0267067)
Supplement: S5 File — (ZIP) [file pone.0267067.s005.zip › mb 1_P-p38_p38-tot Fig 9A/Description of Figure 9A p-38.pdf]

Figure 9A.

The membrane #1 was used for hybridization with anti- phospho p-38 antibody and after stripping the membrane has been hybridized with anti-p38 antibody (details on Mb1 p38).

The order of the sample follow exactly the order on the initial figure presented in the paper and is:
Line 1: control
Line 2: Control + Sb ON
Line 3: ST 1 hour
Line 4: ST 2 hours
Line 5: ST 3 hours
Line 6: ST 1 hours +Sb
Line 7: ST 2 hours+ Sb
Line 8: ST3 hours + Sb
Line 9: SBON+ST 1 hour
Line 10: SbON+ ST 2 hours
Line 11 : SbON+ ST 3hours
