# Supplementary material for: Correction: Interaction of Saccharomyces boulardii with Salmonella enterica Serovar Typhimurium Protects Mice and Modifies T84 Cell Response to the Infection
Source: PLoS One. 2022 Apr 11;17(4):e0267067. doi: 10.1371/journal.pone.0267067 (PMC9000030; doi:10.1371/journal.pone.0267067)
Supplement: S5 File — (ZIP) [file pone.0267067.s005.zip › mbs 4 et 5_ERK-tot_P-ERK_Fig 9A and b-actin for Fig 9A and 10A/Description of Figure 9A-p-ERK.pdf]

Figure 9A.

We have a problem with des-tripping the anti-ERK antibodies (total and phospho) for that reason we use two membranes.

The membrane #4 was used for hybridization with anti-ERK and the membrane #5 was used for hybridization with anti- phospho -ERK and after stripping the membrane has been hybridized with anti-actin antibody (details on M4&5 ERK).

As we load the same protein samples on parallel blots (M#1, M#2, M#3, M#4 and M#5) this actin can be use as loading control for all this blots.

The order of the sample follow exactly the order on the initial figure presented in the paper and is:
Line 1: control
Line 2: Control + Sb ON
Line 3: ST 1 hour
Line 4: ST 2 hours
Line 5: ST 3 hours
Line 6: ST 1 hours +Sb
Line 7: ST 2 hours+ Sb
Line 8: ST3 hours + Sb
Line 9: SBON+ST 1 hour
Line 10: SbON+ ST 2 hours
Line 11 : SbON+ ST 3hours

In your e-mail from 10/31/2019 you raise the question of the use of ERK2 antibodies. The reason was that at that time we dispose in our laboratory the anti-ERK2 antibody from Santa Cruz Ref: Sc-1647 that we have use as control.
