# Supplementary material for: Correction: Interaction of Saccharomyces boulardii with Salmonella enterica Serovar Typhimurium Protects Mice and Modifies T84 Cell Response to the Infection
Source: PLoS One. 2022 Apr 11;17(4):e0267067. doi: 10.1371/journal.pone.0267067 (PMC9000030; doi:10.1371/journal.pone.0267067)
Supplement: S6 File — (ZIP) [file pone.0267067.s006.zip › Description of Figure 9B.pdf]

Figure 9B.

We have a problem with des-tripping the anti-ERK antibodies (total and phospho) for that reason we use two membranes.

The membrane #6 was used for hybridization with anti- phospho -ERK and the membrane #7 was used for hybridization with anti- p38. After des-tripping membrane M6 was probing with anti-p38 antibody and membrane M7 with anti-ERK antibody.

We loaded the same protein samples on parallel blots (M#6 and M#7). However as suggested, we exposed the two blots to anti-actin as loading control.

The order of the sample follow exactly the order on the initial figure presented in the paper and is:

Lane 1: control
Lane 2: ST 3 hours
Lane 3: SbON+ ST 3hours
Lane 4: Sb (HT) +ST 3hours
Lane 5: Sb(GB)+ ST 3hours
Lane 6: Sb(W)+ ST 3hours

In your e-mail from 10/31/2019 you raise the question of the use of ERK2 antibodies. The reason was that at that time we dispose in our laboratory the anti-ERK2 antibody from Santa Cruz Ref: Sc-1647 that we have use as control.
